# 3D-Printed Region-Specific Biomimetic Prosthesis for Enhancing Heterogeneous Double-Interface Integration of Bone–Prosthesis and Tendon–Prosthesis

**DOI:** 10.34133/bmr.0416

**Published:** 2026-09-11

**Authors:** Jinbo Zhang, Qing Han, Xue Zhao, Xingzhen Li, Zhaowei Zhang, Tao Lv, Jincheng Wang, Hao Chen

**Affiliations:** ^1^ Department of Orthopedics, The Second Hospital of Jilin University, Changchun, China.; ^2^ Department of Endocrinology and Metabolism, The First Hospital of Jilin University, Changchun, China.

## Abstract

Large bone defects with loss of natural tendon and soft tissue attachment sites remain challenging in prosthetic reconstruction because both bone–prosthesis and tendon–prosthesis interface integration must be achieved. To address this heterogeneous dual-interface requirement, this study proposes and validates a 3-dimensional printed (3D-printed) region-specific biomimetic titanium alloy prosthesis design strategy. This strategy integrates biomimetic tendon ordered microstructures and biomimetic trabecular-like disordered porous structures within distinct functional regions of the same Ti6Al4V prosthesis. Specifically, the top cap was designed with tendon-like ordered microstructures for soft tissue attachment, whereas the body region was designed with trabecular porous structures for bone integration. In vitro experiments demonstrate that the biomimetic tendon structure markedly promotes the directional alignment and tenogenic differentiation of tendon-derived stem cells, while the biomimetic trabecular-like structure favors osteogenic differentiation and mineralization maturation of bone-marrow-derived mesenchymal stem cells. In vivo animal experiments further validate that a single structure is insufficient to simultaneously meet the dual demands of soft tissue attachment and bone stability. In contrast, the 3D-printed region-specific biomimetic prosthesis establishes spatially partitioned microenvironments for tendon and bone formation in vivo, enabling more coordinated heterogeneous double-interface tissue integration.

## Introduction

In cases of severe bone defects or complex joint reconstruction surgeries, substantial bone loss often results in the loss of natural attachment points for soft tissues such as tendons or ligaments [1]. To maintain the long-term stability of joint prostheses after reconstruction, clinical practice typically requires direct suturing of tendons onto the surface of metal prostheses, rather than achieving classic tendon–bone interface healing [2]. Therefore, prosthetic reconstruction with substantial bone loss inherently faces 2 interrelated but biologically distinct interface challenges. On the bone side, the prosthesis must achieve stable bone–prosthesis integration to bear long-term mechanical loads, while on the soft tissue side, a stable tendon–prosthesis interface must form to maintain the joint’s long-term functional mobility. This necessitates the coordinated integration of a heterogeneous double interface between the bone–prosthesis and tendon–prosthesis interfaces, meaning that the prosthesis system must simultaneously achieve long-term load-bearing stability at the bone side and functional attachment at the soft tissue side [3]. In terms of material selection, titanium alloys, which provide a high elastic modulus, excellent mechanical strength, and long-term structural stability, meet the load-bearing and fatigue resistance requirements of joint reconstruction, making them the irreplaceable material of choice in clinical practice [4,5]. In recent years, with the development of porous structural designs and 3-dimensional-printing (3D-printing) technology, considerable advancements have been made in porous titanium alloy prostheses, promoting bone tissue ingrowth and improving the mechanical stability of the bone–prosthesis interface. However, their microstructural designs often focus primarily on bone integration, making it difficult to simultaneously address the structural and biological needs of both the bone and soft tissue sides [6].

In response to the clinical need for heterogeneous double-interface integration, considerable progress has been made with porous titanium alloy prostheses in promoting bone tissue ingrowth and improving the mechanical stability of the bone–prosthesis interface, with their microporous structural designs primarily focused on bone integration [7]. However, there is a notable deficiency in the optimization of microporous structures for soft tissue attachment, which leads to a clear lack of coordination between the bone and soft tissue interfaces in terms of integration effectiveness [5,8]. Specifically, at the soft tissue interface, the high elastic modulus and relatively biologically inert surface characteristics of titanium alloy prostheses often result in tendon repair predominantly through disordered scar tissue, which hinders the reconstruction of a stable and physiologically functional attachment structure. This, in turn, limits the mechanical properties of the tendon–prosthesis interface [9]. Clinical follow-up data further indicate that in typical clinical scenarios such as rotator cuff repairs, the 5-year revision-free survival rate of soft tissue attachment points on metal prostheses is 74% [10]. However, the incidence of prosthesis failure due to soft tissue dysfunction can exceed 60% [11], highlighting that, even when reliable fixation is achieved at the bone–prosthesis interface, failure in soft tissue attachment remains a critical factor contributing to prosthetic instability and functional impairment.

In an effort to enhance the biological integration of the tendon–prosthesis interface, previous studies have explored various strategies such as surface coatings, bioactive molecule delivery, and growth factor modifications to improve tendon attachment. These approaches include the incorporation of bone morphogenetic protein-2 (BMP-2), transforming growth factor (TGF), or adhesion peptides to promote osteogenesis, tenogenesis, or cell adhesion [12]. While these strategies can regulate cellular responses at the interface and improve early tissue healing, their long-term effectiveness depends heavily on factors like the concentration of growth factors, their release kinetics, and the stability of the local microenvironment. Furthermore, the clinical translation of these strategies remains uncertain [13,14]. More importantly, these methods often treat the prosthesis as a structurally homogeneous functional component, focusing on modulating cellular behavior at a single interface. This approach neglects the unique structural and mechanical characteristics of the natural tendon–bone interface, which is not a uniform connection but a continuous gradient system, where ordered collagen fibers transition into an unordered trabecular bone network [15]. The single-interface design approach struggles to address the distinct biomechanical requirements of both the bone and soft tissue sides, thereby hindering the long-term coordinated stability of the dual interfaces.

Reconstructing the spatial organization of natural tissue interfaces is considered a key approach in biomimetic design for achieving functional integration across multiple tissue interfaces [14]. In recent years, numerous studies have demonstrated the effectiveness of biomimetic structurally organized scaffolds in the repair of typical interfaces, such as cartilage–bone. For example, creating a regionally organized system within a single scaffold that transitions from a low-modulus, porous cartilage-like structure to a high-modulus, mineralized bone-like structure can induce both the formation of hyaline cartilage and the reconstruction of underlying new bone in vivo, markedly reducing stress concentrations at the interface [14,16,17]. Similar region-specific design strategies have been applied to tendon–bone and ligament–bone repair. By arranging ordered fibrous structures and disordered porous structures in different functional regions, these strategies guide cellular alignment on the soft tissue side and promote tissue ingrowth on the bone side, leading to more natural tissue integration that mimics the characteristics of native insertion points in animal models [14]. With advancements in metal 3D-printing technology, this concept of region-specific biomimetic design has been extended from polymer and composite materials to high-modulus metal prostheses. By precisely controlling pore size, porosity, and structural orientation, it is now possible to fabricate multiregion, multiscale microstructures within a single titanium alloy component, providing a solid engineering foundation for incorporating region-specific biomimetic designs into the coordination and integration of heterogeneous double interfaces in prostheses [18,19].

Building on the understanding outlined above, this study proposes a design strategy for a 3D-printed region-specific biomimetic titanium alloy prosthesis aimed at achieving the synergistic integration of heterogeneous bone–prosthesis and tendon–prosthesis interfaces. On the bone side, the prosthesis introduces a biomimetic trabecular bone porous structure to enhance the osteogenic differentiation potential of bone-marrow-derived mesenchymal stem cells (BMSCs) and improve the mechanical stability of the bone–prosthesis interface. On the soft tissue side, a biomimetic tendon-like ordered, streamlined microporous structure is developed to guide the directional alignment of tendon-derived stem cells (TDSCs) and enhance the mechanical performance of the tendon–prosthesis interface. By integrating different biomimetic microstructures within distinct functional regions of the same prosthesis, this study aims to systematically elucidate the effects of structural biomimicry on the regulation of cellular behavior and tissue integration at the heterogeneous double interfaces. To validate this design concept, the study further combines in vitro cell experiments and in vivo animal models to assess, from both biological and mechanical perspectives, the potential advantages of the region-specific biomimetic prosthesis in promoting the synergistic integration of the bone–prosthesis and tendon–prosthesis interfaces.

## Materials and Methods

### Design and fabrication of porous Ti6Al4V scaffolds

In this study, the computer-aided design software Rhinoceros 8 and its parametric modeling plug-in Grasshopper (McNeel, Seattle, WA, USA) were used to design the porous Ti6Al4V scaffolds. The parameters for the porous structure were determined based on previous research by our group, with the pore size set at 500 μm and a total porosity of approximately 60% [19,20]. Preliminary studies have shown that under these pore size and porosity conditions, the porous scaffolds achieve optimal cell adhesion and proliferation. To eliminate potential interference from variations in pore size and porosity, scaffolds for all experimental groups were designed with similar pore structural parameters. For different experimental needs, scaffolds were designed for in vitro and in vivo studies. The in vitro experiment used a disc-shaped scaffold with a diameter of 10 mm and a thickness of 1.5 mm, while the in vivo experiment used a nail-shaped scaffold with a diameter of 5 mm and a height of 8 mm, including the biomimetic tendon (BTen), biomimetic trabecular bone (BT), and biomimetic region-specific (BR) groups. All scaffolds were manufactured using selective laser melting (SLM) equipment (BLT-A3200, Germany). The printing material was Ti6Al4V titanium alloy powder (TLS, Germany), with a powder particle size range of 45 to 100 μm. After printing, all scaffolds underwent ultrasonic cleaning in ethanol and deionized water for 60 min to remove any residual unmelting powder from the pores. Subsequently, the scaffolds were sterilized in a high-pressure steam autoclave (120 °C for 30 min) and dried in an oven at 60 °C to remove any remaining moisture in the porous structure. Finally, the scaffolds were further disinfected under ultraviolet light for 60 min before being used for subsequent in vivo and in vitro experiments.

### Material characterization of porous scaffolds

The microstructure, surface morphology, and chemical composition of the porous Ti6Al4V scaffolds were systematically characterized using scanning electron microscopy (SEM; GeminiSEM 300, ZEISS, Germany) and energy-dispersive x-ray spectroscopy (EDS; Xplore 30, Oxford Instruments, UK). Prior to testing, all scaffold samples were ultrasonically cleaned and then coated with a thin layer of gold using a metal sputter coater to enhance their electrical conductivity. SEM images were subsequently acquired at magnifications of 50× and 1,000× under an accelerating voltage of 10 to 15 kV, allowing for the observation of pore morphology, pore size, and spatial distribution. Additionally, EDS was used for elemental analysis to record the distribution and relative content of the main alloying elements, verifying that the material composition met the design expectations for Ti6Al4V alloy.

### Computational fluid dynamics analysis of permeability

The fluid transport performance of the BTen and BT titanium alloy scaffolds was evaluated using computational fluid dynamics (CFD) simulations. Scaffold models were imported into ANSYS Fluent and discretized with high-density meshes. Because the Reynolds number was <1, a laminar flow model was applied. The fluid phase was defined as Dulbecco’s modified Eagle medium, with a density of 1,000 kg/m^3^ and a dynamic viscosity of 1.45 × 10^−3^ Pa·s. Boundary conditions included an inlet velocity of 0.001 m/s, a zero-pressure outlet, and no-slip conditions at the scaffold walls [21]. The pressure drop (Δ*p*) was defined as the difference between inlet and outlet pressures, with *L* denoting the model height along the flow direction. Scaffold permeability was calculated according to Darcy’s law [21]:

$$K_{C}=\frac{\mu \mathrm{QL}}{A∆p}$$ (1)

where *K_C_* is the permeability, *μ* is the dynamic viscosity, *Q* is the volumetric flow rate, *L* is the model height, *A* is the cross-sectional area perpendicular to the flow direction, and Δ*p* is the pressure drop. To compare the permeability between the 2 structures, the calculated permeability values were normalized to the BT group and expressed as relative permeability.

### In vitro cell experiments

#### Biocompatibility evaluation

The porous scaffolds from each group were placed in 48-well plates. TDSCs were seeded onto the surface of the biomimetic tendon (BTen) scaffolds, and BMSCs were seeded onto the biomimetic trabecular (BT) scaffolds, at a density of 5 × 10^4^ cells/well. The cells were cultured at 37 °C with 5% CO_2_, and the medium was changed every 24 h.

##### CCK-8 cell proliferation assay

Cell proliferation on the scaffold surfaces was quantitatively assessed using the Cell Counting Kit-8 (CCK-8) method (Bioss, China). Each group had 6 scaffolds, with 3 scaffolds each receiving 1 ml of TDSC or BMSC suspension (5 × 10^4^ cells/ml). After culturing for 1, 3, and 5 d, CCK-8 assays were performed. The absorbance at 450 nm was measured using a microplate reader (Bio-Rad, CA, USA), and the data were analyzed and graphed using GraphPad Prism 9.0.

##### Live/dead staining

Cell viability was qualitatively assessed using a live/dead staining kit (BestBio, China). The experimental setup and cell seeding density were the same as in the CCK-8 assay. After 1, 3, and 5 d of culture, cells were stained following the kit protocol and observed under a fluorescence microscope (Olympus, Japan). The ImageJ software was used for the semiquantitative analysis of live/dead cell distribution.

##### Cell adhesion and morphology

To assess cell adhesion and cytoskeletal morphology, scaffolds with TDSCs or BMSCs were cultured for 7 d. After washing with phosphate-buffered saline (PBS), cells were fixed with 4% paraformaldehyde (PFA) for 10 min and permeabilized with 0.1% Triton X-100. Rhodamine–phalloidin staining (1:200) was used to visualize the cytoskeleton, followed by 4′,6-diamidino-2-phenylindole (DAPI) nuclear staining (1:500). The samples were observed under a fluorescence microscope, with rhodamine–phalloidin staining showing the cytoskeleton in green and DAPI labeling the nuclei in blue. For quantitative analysis of cell number, DAPI-positive nuclei were counted using the ImageJ software from randomly selected microscopic fields under the same magnification and imaging parameters. Micro-computed tomography (micro-CT) reconstruction was used to confirm comparable external dimensions and projected observation areas between the BTen and BT scaffolds. To avoid bias caused by differences in internal pore wall surface area between different porous architectures, the internal pore surface area was excluded from normalization.

#### Evaluation of tenogenic differentiation

TDSCs were seeded onto the scaffold surfaces at a density of 1 × 10^5^ cells/well in 48-well plates. The cells were initially cultured in basal medium for 3 d and then switched to tenogenic differentiation medium (ShareBio, China), with the medium changed every 48 h. After 7 or 14 d of induction, the tenogenic differentiation capacity of TDSCs on the scaffolds was evaluated.

##### Immunofluorescence

After culturing TDSCs on scaffolds for 7 d at 37 °C with 5% CO_2_, immunofluorescence staining was used to detect the expression of tenogenic markers. The cells were fixed with 4% PFA for 20 min, permeabilized with 0.1% Triton X-100, and incubated with primary antibodies against collagen I (COL I), scleraxis (SCX), tenomodulin (TNMD), and tenascin-C (TNC) (1:300 dilution) overnight at 4 °C. After washing, secondary antibodies (1:300 dilution) were added and incubated for 30 min. DAPI staining was used to visualize cell nuclei. The samples were observed under a fluorescence microscope (Olympus, Japan), with COL I and TNMD showing green fluorescence and SCX and TNC showing red fluorescence. For quantitative analysis of cell number, DAPI-positive nuclei were counted using the ImageJ software from 20 randomly selected fields in each group under the same magnification and imaging parameters. The results are expressed as the number of DAPI-positive nuclei per field.

##### Enzyme-linked immunosorbent assay

After 7 d of co-culture at 37 °C with 5% CO_2_, the culture supernatant was collected for enzyme-linked immunosorbent assay (ELISA) detection of tenogenic markers, including COL I, SCX, TNMD, and TNC. The assay was performed according to the kit instructions, with the absorbance measured at 450 nm. The concentration of target proteins was calculated using a standard curve. After supernatant collection, cells remaining on the scaffolds were lysed, and the total cellular protein concentration of each corresponding sample was measured using the bicinchoninic acid (BCA) protein assay according to the manufacturer’s instructions. To minimize the influence of differences in cell number among groups, ELISA results were normalized to the corresponding total cellular protein content and expressed as target protein level per milligram of total cellular protein. Data were analyzed using GraphPad Prism 9.0.

##### Real-time quantitative PCR

After co-culturing TDSCs on scaffolds for 7 and 14 d, real-time quantitative polymerase chain reaction (RT-qPCR) was used to assess the expression of tenogenic-related genes, including COL I, SCX, TNMD, and TNC, with GAPDH as the internal control. The qPCR reaction was performed using BlazeTaq SYBR Green qPCR Mix 2.0 (GeneCopoeia, USA) on a LightCycler 480 PCR system (Roche Diagnostics, Switzerland). The primer sequences of each gene are shown in Table S1. The Ct values were analyzed using the GraphPad Prism 9.0 software to compare gene expression levels across different scaffold groups.

#### Evaluation of osteogenic differentiation

BMSCs were seeded onto the surface of the scaffolds at a density of 1 × 10^5^ cells/well in 48-well plates. The cells were initially cultured in basal medium for 3 d and then switched to osteogenic induction medium (basal medium supplemented with 10 mmol/l β-glycerophosphate, 10 nmol/l dexamethasone, and 50 μg/ml ascorbic acid) with medium changes every 48 h. After 7 or 14 d of induction, the osteogenic differentiation capacity of BMSCs on the scaffolds was evaluated.

##### Alkaline phosphatase activity and staining

Alkaline phosphatase (ALP) activity was assessed using the BCIP/NBT staining kit (Beyotime, China). After 7 and 14 d of osteogenic induction, scaffolds were fixed with 4% PFA for 10 min and stained with the color reagent. ALP activity was quantified using the ALP activity detection kit (Beyotime, China), and the absorbance was measured at 450 nm.

##### Alizarin Red S staining and quantification

Alizarin Red S (ARS) staining was used to evaluate the formation of mineralized nodules. After washing with PBS and fixing with 4% PFA for 10 min, scaffolds were stained with ARS for 30 min. Mineralized nodules were quantified by dissolving them with 10% hexadecylpyridinium chloride and measuring the absorbance at 562 nm.

##### Immunofluorescence

After 7 d of co-culture, immunofluorescence staining was performed to detect osteogenic markers, including runt-related transcription factor 2 (RUNX2), osteocalcin (OCN), decorin (DCN), and BMP-2. Cells were fixed, permeabilized, and incubated with primary antibodies (1:300 dilution) overnight at 4 °C. After washing, secondary antibodies (1:300 dilution) were added, followed by DAPI staining for nuclei. Fluorescence was observed under a microscope (Olympus, Japan), with RUNX2 and DCN showing green fluorescence and OCN and BMP-2 showing red fluorescence. DAPI-positive nuclei were quantified using the ImageJ software from 20 randomly selected fields in each group under the same magnification and imaging parameters.

##### Enzyme-linked immunosorbent assay

After 7 d of co-culture, culture supernatants were collected for ELISA detection of osteogenic markers, including RUNX2, OCN, DCN, and BMP-2, using the corresponding ELISA kits. The absorbance was measured at 450 nm, and protein concentrations were calculated from standard curves. After supernatant collection, cells remaining on the scaffolds were lysed, and the total cellular protein concentration of each corresponding sample was measured using the BCA protein assay according to the manufacturer’s instructions. To minimize the influence of differences in cell number among groups, ELISA results were normalized to the corresponding total cellular protein content and expressed as target protein level per milligram of total cellular protein. Data were analyzed using GraphPad Prism 9.0.

##### Real-time quantitative PCR

After 7 and 14 d of co-culture, RT-qPCR was performed to assess the expression of osteogenic-related genes (RUNX2, OCN, DCN, and BMP-2), with GAPDH as the internal control. qPCR reactions were performed using BlazeTaq SYBR Green qPCR Mix 2.0 (GeneCopoeia, USA) and analyzed on a LightCycler 480 PCR system (Roche Diagnostics, Switzerland). The primer sequences of each gene are shown in Table S1. The relative gene expression levels were compared across different scaffold groups using GraphPad Prism 9.0.

### In vivo experiments

#### Animal experiment

A total of 36 male New Zealand rabbits were randomly divided into 3 groups, with 12 rabbits per group. Prior to surgery, general anesthesia was induced by intramuscular injection of xylazine and ketamine, followed by shaving and disinfecting the right knee joint area with iodine tincture. Local infiltration anesthesia was administered during the procedure. A 5-cm longitudinal incision was made along the knee joint, and the fascia was separated to expose the patellar tendon. The patellar tendon was then severed at its attachment to the proximal tibia, and the tendon stump was sutured to the upper end of the prosthesis using absorbable sutures. A 5-mm-diameter bone tunnel was created in the proximal tibia using a dental drill, and the lower end of the prosthesis was inserted and fixed within the tunnel. The surgical site was thoroughly irrigated and closed in layers. Postoperatively, penicillin was administered via intramuscular injection daily for infection prevention for the first 3 d. The experimental procedures were approved by the Animal Ethics Committee of Jilin University and followed relevant ethical guidelines.

#### Micro-CT evaluation

At the 6th and 12th weeks postimplantation, the animals were euthanized using carbon dioxide, and the proximal tibia samples were immediately fixed in 4% PFA. The samples were then scanned using a micro-CT scanner (VNC-102), and the images of new bone formation and scaffold structure were reconstructed for visualization. A cylindrical region containing the scaffold was defined as the region of interest, and micro-CT data were used to quantitatively analyze bone formation, including bone volume/total volume ratio (BV/TV), trabecular separation (Tb.Sp), and trabecular thickness (Tb.Th).

#### Histological evaluation

After collection, tibia samples were fixed in 4% PFA and dehydrated through a graded ethanol series. The samples were then embedded in resin (Heraeus, Germany) using a light-curing device and sectioned into 200-μm-thick slices using an EXAKT 300CP microtome (EXAKT, Germany). To evaluate tendon ingrowth and tenogenic differentiation, sections were stained with hematoxylin and eosin (HE) and Sirius Red. The stained slices were observed using an optical and confocal microscope for both qualitative and quantitative analyses. For immunofluorescence, samples were fixed in 4% PFA for 4 h, dehydrated, embedded, and sectioned into 5-μm slices. Antigen retrieval was performed with citrate buffer. Sections were blocked with PBS containing 5% normal goat serum and 0.3% Triton X-100, followed by incubation with primary antibodies against tenogenic (COL I and SCX) and osteogenic (RUNX2 and OCN) markers (1:200 dilution) overnight at 4 °C. After washing, secondary antibodies (1:500 dilution) were applied, followed by DAPI nuclear staining. The expression and distribution of the markers were analyzed using fluorescence microscopy.

#### Mechanical pullout testing

To evaluate the functional mechanical stability of the tendon–prosthesis interface, pullout testing was performed at 6 and 12 weeks after implantation. After euthanasia, the tendon–prosthesis complexes were carefully harvested while preserving the tendon attachment region. The specimens were fixed on a mechanical testing machine, with the tendon and prosthesis secured in opposing clamps and aligned along the tensile loading direction. A uniaxial tensile load was then applied at a displacement rate of 5 mm/min until failure. The maximum pullout force during testing was recorded as the load-to-failure value of the tendon–prosthesis interface. The results were analyzed using GraphPad Prism 9.0.

### Statistical analysis

Data are presented as mean ± standard deviation. Differences between multiple groups were analyzed using one-way analysis of variance. All statistical analyses were performed using the GraphPad Prism 9 software. Statistical significance was defined as a *P* value less than 0.05.

## Results

### Surface morphology and composition of porous Ti6Al4V scaffolds

**Fig. 1.**
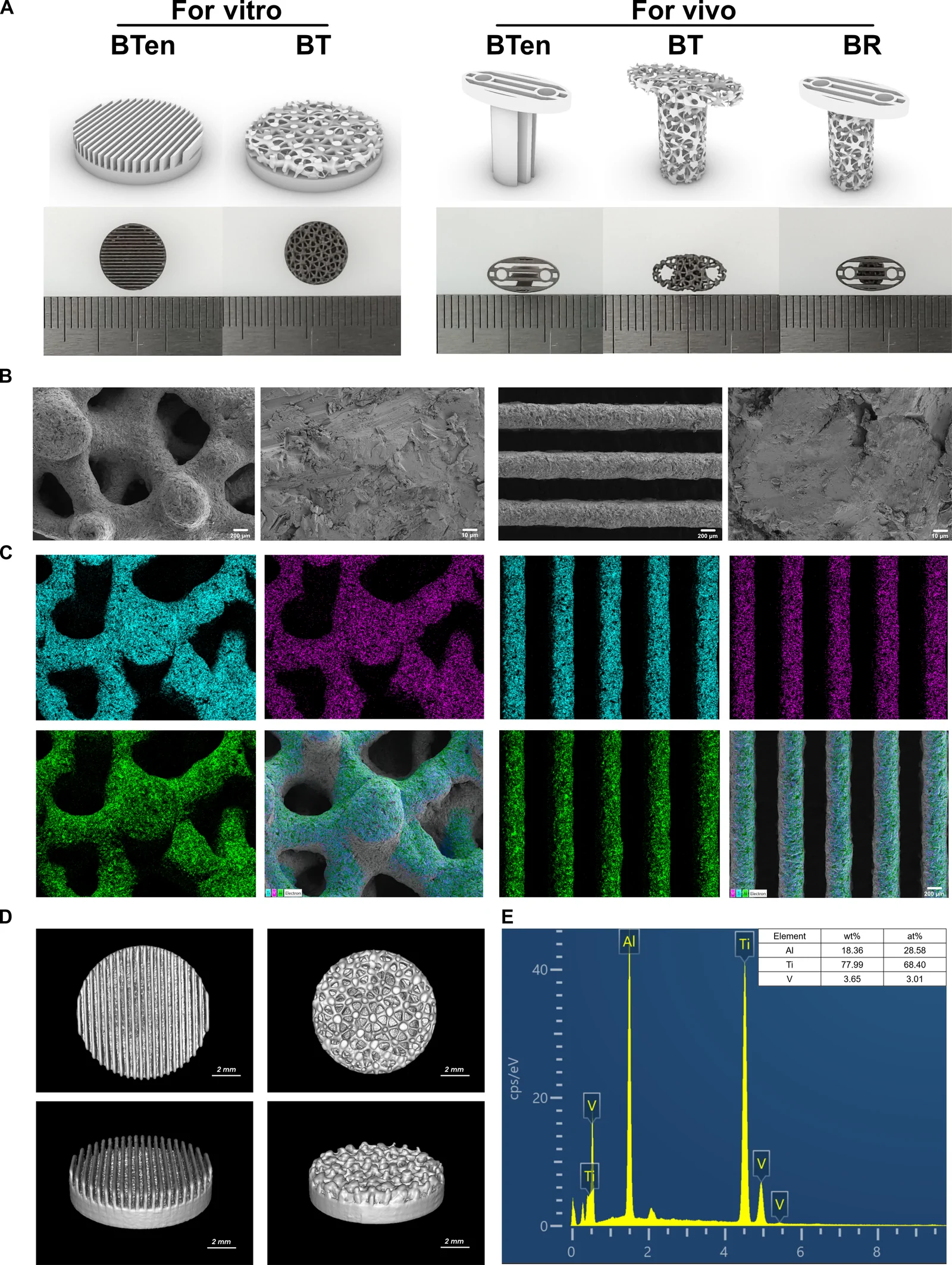
Scaffold morphology and composition characterization. (A) Computer-aided design (CAD) and gross appearance images of Ti6Al4V scaffolds used for in vitro and in vivo experiments. Cylindrical biomimetic tendon micropore scaffolds (BTen) and biomimetic trabecular scaffolds (BT) were used for in vitro cell experiments, while nail-shaped BTen, BT, and BR scaffolds were used for in vivo implantation experiments. (B) Scanning electron microscopy (SEM) images showing the surface morphology and porous architecture of the BT and BTen scaffolds. The BT scaffold exhibited a randomly interconnected trabecular-like porous structure, whereas the BTen scaffold showed an ordered parallel microporous structure. (C) Energy-dispersive x-ray spectroscopy (EDS) elemental mapping images showing the distribution of the Ti, Al, and V elements on the BT and BTen scaffold surfaces. (D) Micro-computed tomography (micro-CT) reconstructed images showing the 3-dimensional porous architectures of the BTen and BT scaffolds. (E) Representative EDS spectrum and quantitative elemental composition analysis of the Ti6Al4V scaffold.

This study designed 2 scaffold specifications for in vitro cell and in vivo experiments based on previous research and experimental requirements. As shown in Fig. 1A, cylindrical scaffolds with a diameter of 10 mm and a height of 1.5 mm were used for in vitro cell experiments, while nail-shaped scaffolds with a top cap long diameter of 10 mm, a short diameter of 5 mm, and a height of 8 mm were used for in vivo experiments. Porous Ti6Al4V scaffolds were successfully fabricated using SLM, and the printed scaffolds were generally consistent with the computer-aided designs. SEM analysis showed that the BTen scaffold exhibited an ordered parallel microporous structure, whereas the BT scaffold showed a randomly interconnected trabecular bone-like structure. The actual pore sizes of the BTen and BT scaffolds were 472 ± 29 and 459 ± 35 μm, respectively, with corresponding porosities of 56.8% ± 1.9% and 54.6% ± 2.4% (Table S2 and Fig. S1). Both scaffolds showed rough surfaces with partially adhered powder particles, which are typical features of SLM-fabricated titanium alloy scaffolds (Fig. 1B). Micro-CT reconstruction further confirmed the 3D porous architectures of the BTen and BT scaffolds (Fig. 1D). EDS elemental mapping and spectrum analysis showed that the scaffolds were mainly composed of Ti, Al, and V, with no obvious elemental contamination during fabrication (Fig. 1C and E).

### Biocompatibility evaluation of scaffolds

**Fig. 2.**
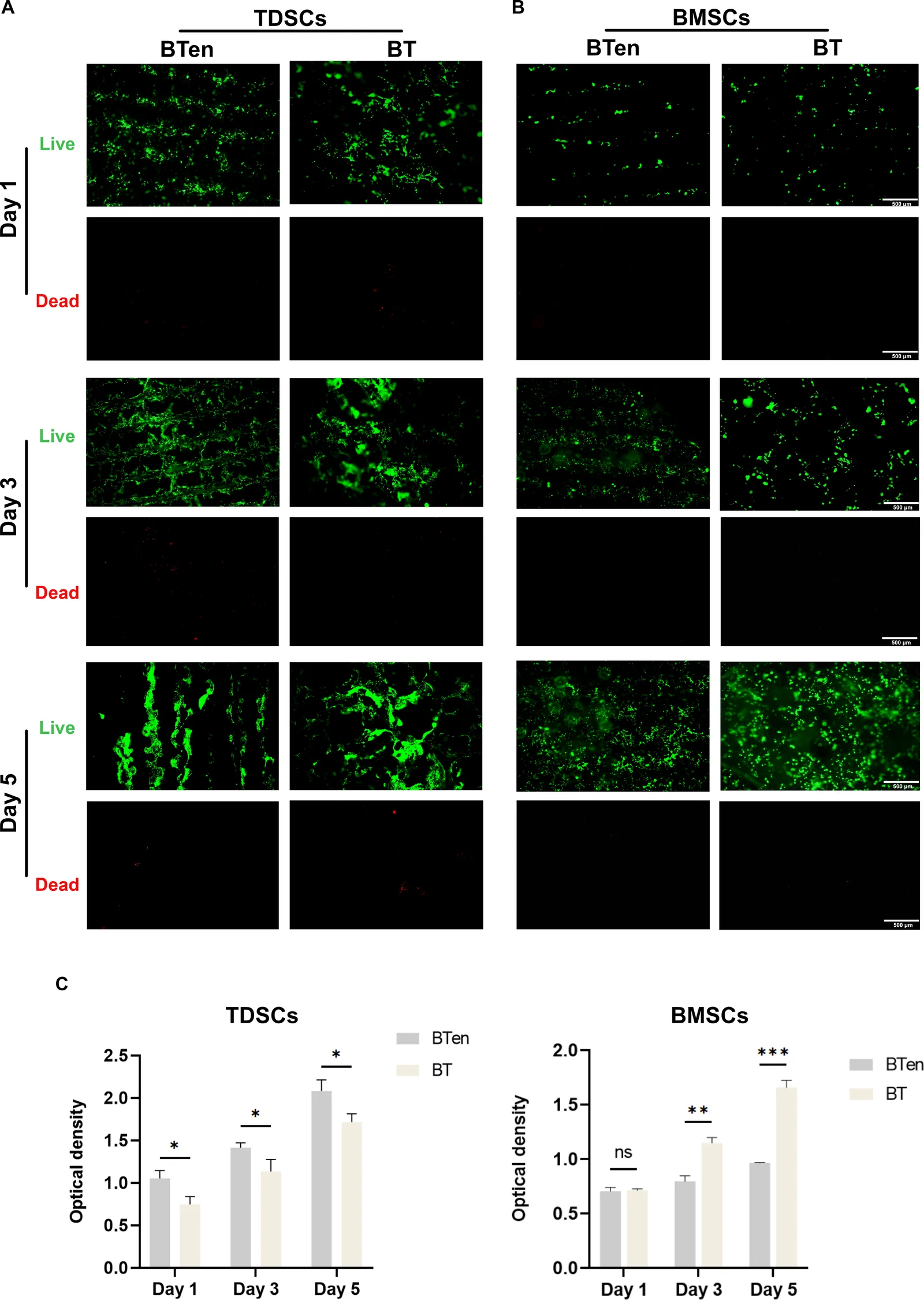
Live/dead staining and proliferation assessment of tendon-derived stem cells (TDSCs) on scaffolds. (A) Live/dead cell fluorescence imaging of TDSCs co-cultured on BTen and BT scaffolds for 1, 3, and 5 d. (B) Live/dead cell fluorescence imaging of bone-marrow-derived mesenchymal stem cells (BMSCs) co-cultured on BTen and BT scaffolds for 1, 3, and 5 d. Green represents live cells; red represents dead cells. (C) Cell Counting Kit-8 (CCK-8) assay for assessing cell proliferation in each group. Multiple comparisons: ns denotes no statistical significance; *P < 0.05, **P < 0.01, and ***P < 0.001.

Before scaffold co-culture experiments, the isolated TDSCs and BMSCs were confirmed to exhibit typical mesenchymal stem cell characteristics, including spindle-like morphology, high expression of CD44 and CD90, low expression of CD31 and CD45, and multilineage differentiation potential (Fig. S2) [22]. To evaluate the biocompatibility of different biomimetic porous scaffolds, BTen and BT scaffolds were co-cultured with TDSCs and BMSCs in vitro. Considering that scaffold permeability may also influence nutrient transport, local fluid microenvironment, and subsequent cell behavior, CFD analysis was further performed to compare the fluid transport characteristics of the BTen and BT structures. Although the 2 structures had similar porosity, the BT structure showed higher relative permeability, whereas the BTen structure exhibited a higher pressure difference under the same flow conditions. The wall shear stress distribution also differed between the 2 scaffolds, indicating that the distinct pore architectures generated different local fluid mechanical microenvironments (Fig. S3). Live/dead fluorescence staining and CCK-8 cell viability assays were performed on days 1, 3, and 5 of culture. Figure 2A and B show the live/dead staining results. Throughout the culture period, both scaffolds primarily exhibited green fluorescence, with minimal red fluorescence, indicating good cell compatibility and no significant time-dependent cytotoxic accumulation. Although overall cell survival rates were similar, differences in cell growth were observed between the 2 scaffold types.

Figure 2B shows that in the TDSC co-culture, the BTen scaffold exhibited a higher cell adhesion density early in the culture. By day 3, TDSCs on the BTen scaffold spread further, with elongated spindle-shaped cells aligning along the scaffold structure. By day 5, the BTen group formed a continuous and dense cell layer. In contrast, Fig. 2A shows a more sparse distribution of TDSCs on the BT scaffold. Although cell numbers increased over time, the overall spread and density were lower compared to those of the BTen group. Conversely, in the BMSC co-culture, the BT scaffold showed better support for cell growth. BMSCs in the BT group were evenly distributed, with cell number per microscopic field increasing over time, forming a dense 3D cell network by day 5. On the BTen scaffold, BMSCs exhibited limited adhesion and proliferation, with a significantly lower coverage compared to that of the BT group. Figure 2C shows that CCK-8 analysis results were consistent with the live/dead staining observations. In the TDSC group, the cell activity on the BTen scaffold was higher than that on the BT scaffold on days 1, 3, and 5, with differences increasing over time, suggesting that the biomimetic tendon structure better supports TDSC proliferation. In contrast, BMSCs showed a significantly higher cell activity on the BT scaffold from day 3, with a highly significant difference by day 5, indicating the superior support of the biomimetic trabecular structure for BMSC growth. Together with the CFD results, these findings suggest that the differences in cell growth between the BTen and BT scaffolds may be associated not only with pore size and porosity but also with structure-dependent permeability and local fluid transport characteristics.

**Fig. 3.**
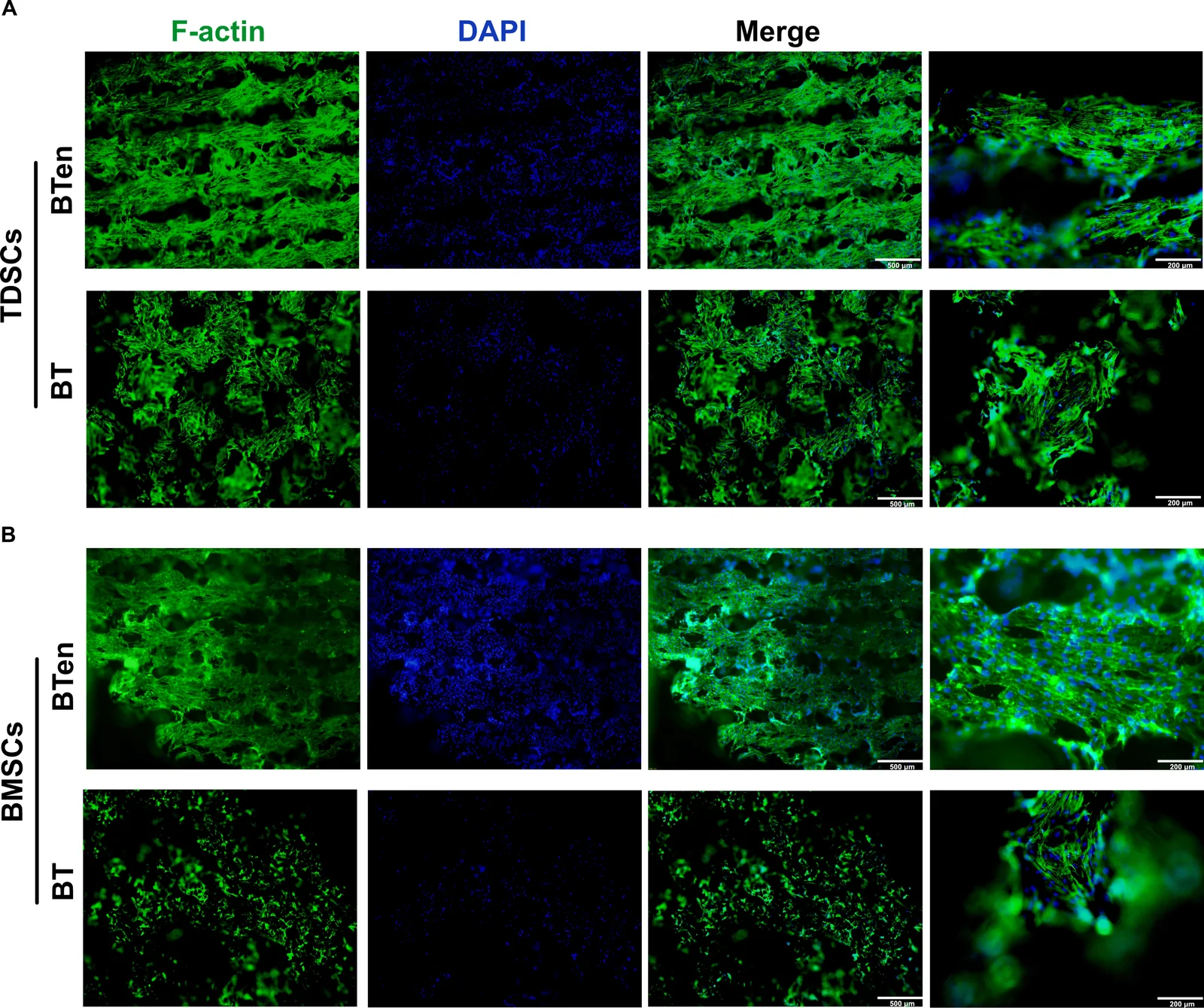
Cytoskeletal assessment on scaffolds. (A) Fluorescence microscopy images of F-actin (green) and 4′,6-diamidino-2-phenylindole (DAPI; blue) staining, showing the cell attachment and cytoskeletal morphology of tendon-derived stem cells (TDSCs) on BTen and BT scaffolds. (B) Fluorescence microscopy images of F-actin (green) and DAPI (blue) staining, showing cell attachment and cytoskeletal morphology of bone-marrow-derived mesenchymal stem cells (BMSCs) on BTen and BT scaffolds.

Additionally, F-actin/DAPI staining was used to observe the cell morphology and cytoskeletal organization on the different scaffolds. Figure 3A shows that in the TDSC co-culture, cells on the BTen scaffold exhibited an elongated spindle shape, with clear F-actin stress fibers aligned along the scaffold’s ordered structure, showing consistent directional distribution. In contrast, TDSCs on the BT scaffold displayed a more irregular cell shape, with scattered F-actin and less pronounced alignment. In the BMSC co-culture, the BT scaffold showed a more uniform and dense cell distribution, with F-actin forming a 3D interconnected network. On the BTen scaffold, BMSCs also aligned to some degree along the ordered structure, but their alignment and cytoskeletal extension were weaker compared to those of TDSCs (Fig. 3B).

### In vitro evaluation of tenogenic differentiation

**Fig. 4.**
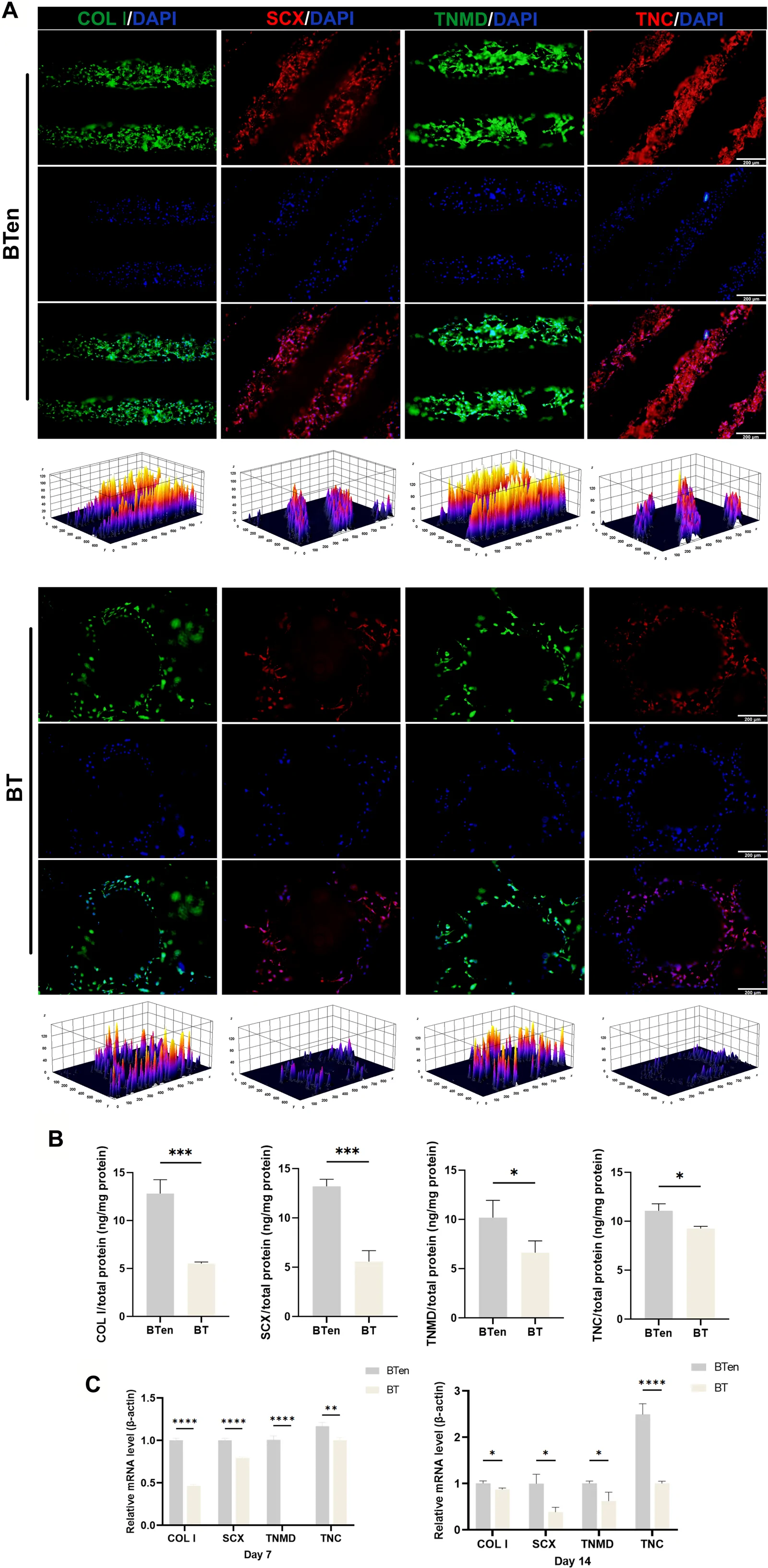
Tenogenic marker expression of tendon-derived stem cells (TDSCs) co-cultured on BTen and BT scaffolds. (A) Immunofluorescence staining showing the protein expression of TDSCs after co-culture for 7 d on BTen and BT scaffolds. (B) Enzyme-linked immunosorbent assay (ELISA) quantification of collagen I (COL I), scleraxis (SCX), tenomodulin (TNMD), and tenascin-C (TNC) concentrations (ng/ml) in the cell supernatant of each group after 7 d. (C) Real-time quantitative polymerase chain reaction (RT-qPCR) analysis of the gene expression of TDSCs on BTen and BT scaffolds at days 7 and 14: COL I, SCX, TNMD, and TNC. Multiple comparisons: *P < 0.05, **P < 0.01, ***P < 0.001, and ****P < 0.0001.

To systematically evaluate the effect of different biomimetic microstructured scaffolds on tendon phenotype formation, TDSCs were co-cultured with scaffolds, and on day 7, immunofluorescence staining and ELISA were performed to analyze the protein expression of key tenogenic markers: COL I, SCX, TNMD, and TNC. Additionally, gene transcription levels were measured on days 7 and 14. On day 7, the immunofluorescence results showed that the BTen group exhibited more pronounced tenogenic phenotype characteristics. Figure 4A demonstrates strong and continuous fluorescence signals for COL I, SCX, TNMD, and TNC, with the proteins distributed in bands or sheets along the scaffold surface, aligning well with the cell orientation, suggesting a typical tendon-like tissue structure. In contrast, the BT group showed weaker fluorescence signals, with dispersed and focal expression regions, making it difficult to form continuous tenogenic structures. DAPI staining further showed the distribution of cell nuclei on the scaffold surface. Quantitative analysis of DAPI-positive nuclei indicated a higher number of TDSCs in the BTen group than in the BT group, suggesting that the BTen structure supported better TDSC adhesion and/or proliferation (Fig. S4). To minimize the influence of differences in cell number on the interpretation of tenogenic differentiation, ELISA results were further normalized to the corresponding total cellular protein content measured by BCA assay (Fig. S5). After normalization, the BTen group still showed significantly higher levels of COL I, SCX, TNMD, and TNC than the BT group. Notably, SCX and TNMD, key markers of tendon lineage commitment and maturation [23], remained notably up-regulated on the BTen scaffold, indicating that the biomimetic tendon-like structure effectively induced TDSCs to differentiate toward the tendon phenotype at an early stage rather than merely increasing cell abundance.

At the transcriptional level, qPCR results revealed a time-dependent structural effect. On day 7, the messenger RNA (mRNA) expression levels of COL I, SCX, TNMD, and TNC were significantly higher in the BTen group than in the BT group. By day 14, these differences were maintained, and some genes, particularly TNMD and TNC, showed an even greater expression gap, suggesting that the tenogenic phenotype on the BTen scaffold was continuously strengthened and stabilized over time (Fig. 4B).

In summary, combining the protein expression results on day 7 with gene expression analysis on days 7 and 14, the BTen scaffold significantly enhanced the expression of tenogenic markers at multiple time points. This suggests that the BTen scaffold not only induces early activation of tendon-related genes but also sustains and strengthens the tenogenic phenotype in the midterm, providing a favorable structural foundation for stable tendon-like tissue formation.

### In vitro osteogenic capacity evaluation

To systematically evaluate the impact of different biomimetic microstructured scaffolds on osteogenic differentiation, BMSCs were co-cultured with BTen and BT scaffolds, and the analysis covered early osteogenic activity, mineralization ability, and osteogenic marker expression.

**Fig. 5.**
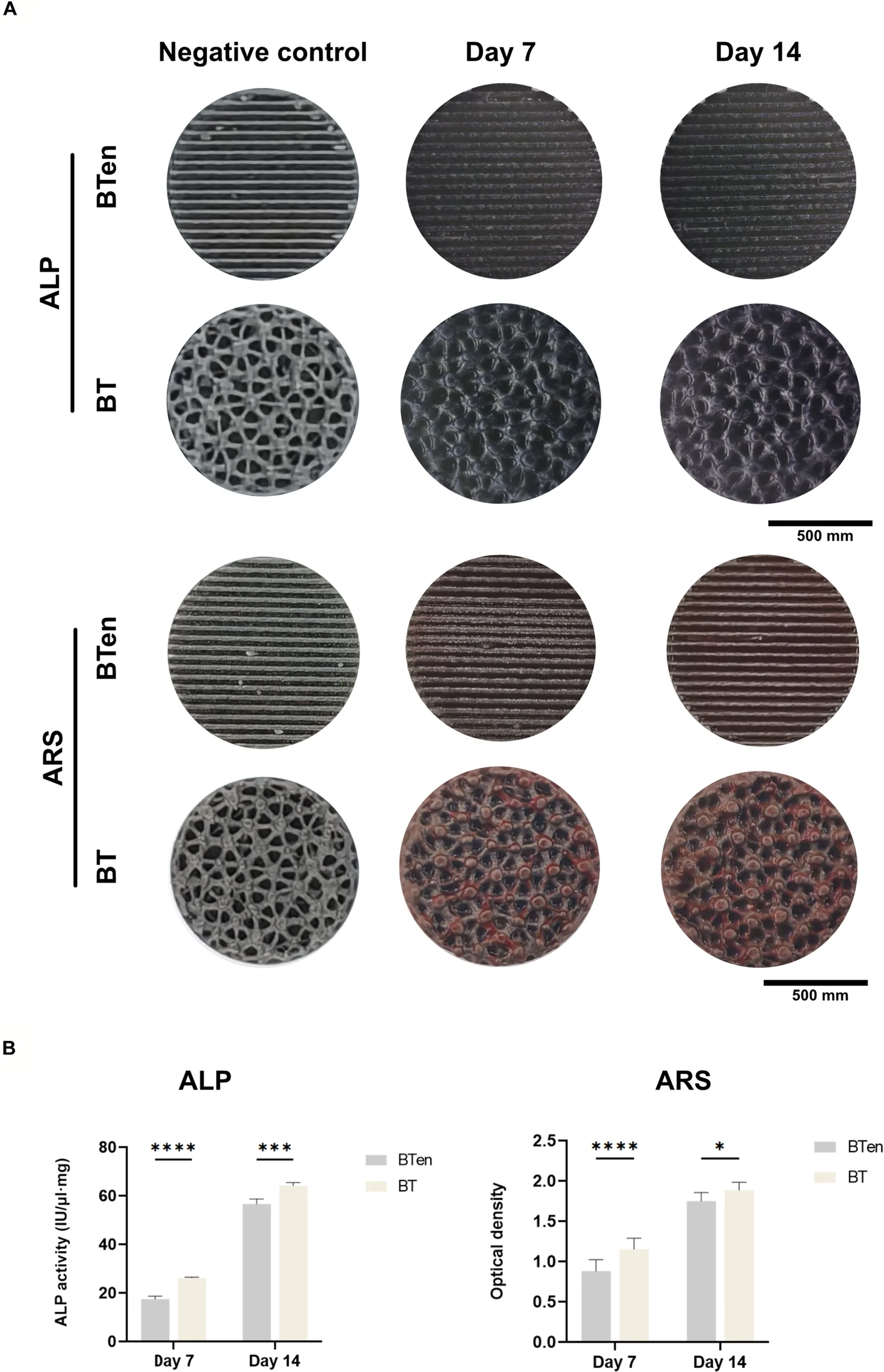
Osteogenic evaluation of bone-marrow-derived mesenchymal stem cells (BMSCs) on BTen and BT scaffolds. (A) Alkaline phosphatase (ALP) and Alizarin Red S (ARS) staining results of BMSCs co-cultured on BTen and BT scaffolds for 7 and 14 d. (B) Quantitative analysis of ALP and ARS results for BMSCs co-cultured on BTen and BT scaffolds for 7 and 14 d. *P < 0.05, ***P < 0.001, and ****P < 0.0001.

Figure 5A shows the ALP staining results on days 7 and 14. The BT group exhibited stronger and more continuous ALP staining at both time points. On day 7, BT scaffolds showed more uniform and continuous ALP staining, while the BTen group displayed weaker staining with more localized positive regions. By day 14, both groups showed enhanced ALP staining, but the BT group showed a more pronounced increase. Figure 5B shows the corresponding quantitative ALP activity analysis, with the BT group showing markedly higher ALP levels on both days 7 and 14, and the difference further widening over time. To further assess mineralization, ARS staining was performed. On day 7, BT scaffolds exhibited clear calcium deposition signals, while mineralization in the BTen group remained limited. By day 14, the BT group showed a significant increase in mineralized nodules, with denser deposition covering a larger area, exhibiting typical bone-like mineralization. The BTen group also showed increased mineralization, but overall staining intensity and deposition density were lower than in the BT group (Fig. 5A). Quantitative ARS analysis was consistent with the staining observations, with the BT group showing higher mineralization levels at both time points (Fig. 5B).

**Fig. 6.**
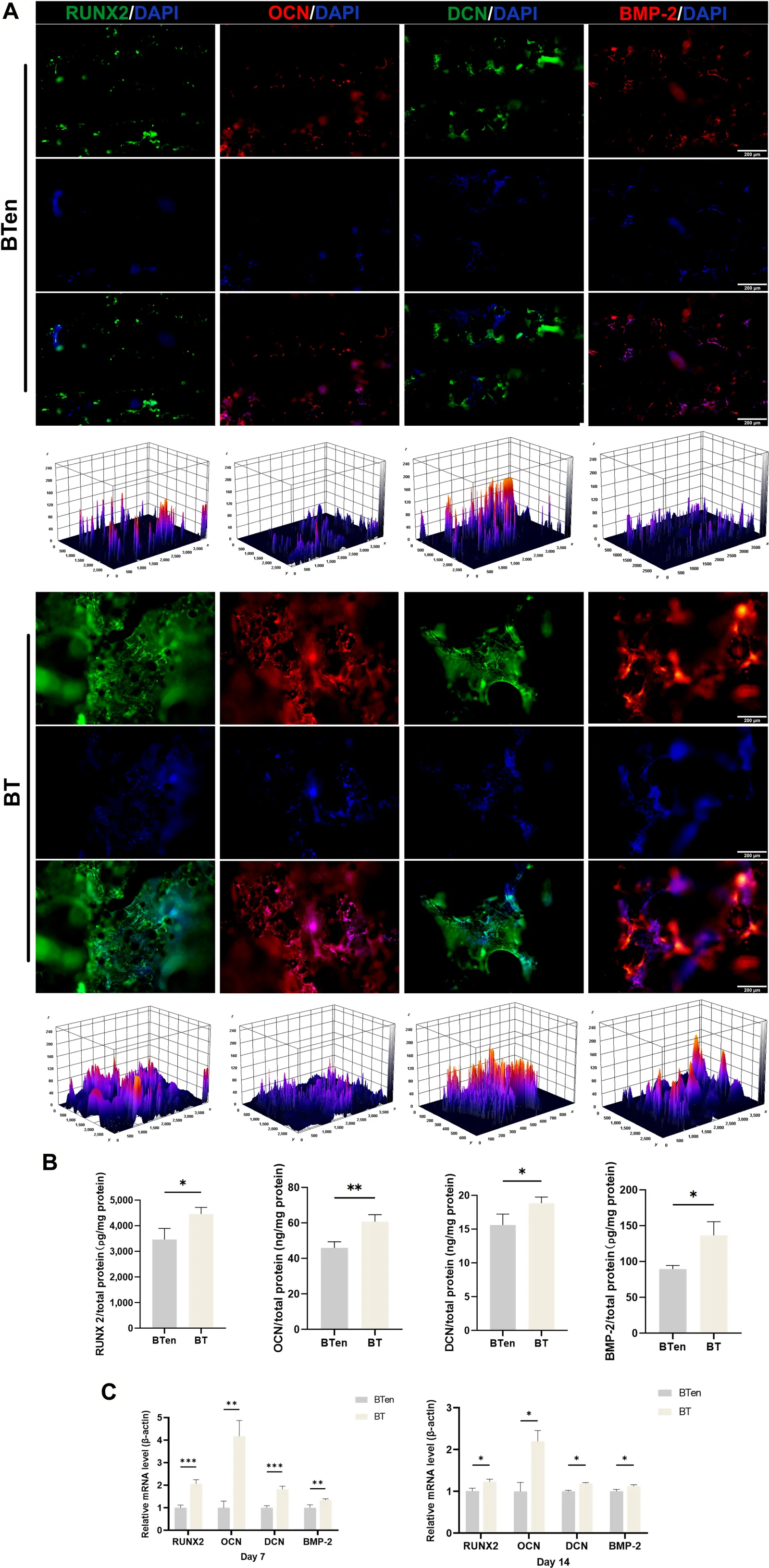
Osteogenic markers of bone-marrow-derived mesenchymal stem cells (BMSCs) co-cultured on BTen and BT scaffolds. (A) Immunofluorescence staining showing the osteogenic marker protein expression of BMSCs after co-culture for 7 d on BTen and BT scaffolds. (B) Enzyme-linked immunosorbent assay (ELISA) quantification of runt-related transcription factor 2 (RUNX2), osteocalcin (OCN), decorin (DCN), and bone morphogenetic protein-2 (BMP-2) concentrations in the cell supernatant of each group after 7 d. (C) Real-time quantitative polymerase chain reaction (RT-qPCR) analysis of gene expression in BMSCs on BTen and BT scaffolds at days 7 and 14: collagen I (COL I), scleraxis (SCX), tenomodulin (TNMD), and tenascin-C (TNC). Multiple comparisons: *P < 0.05, **P < 0.01, and ***P < 0.001.

To further confirm the scaffold’s role in regulating osteogenic phenotype at the molecular level, immunofluorescence staining of key osteogenic markers was performed on day 7. Figure 6A shows that in the BT group, RUNX2, OCN, DCN, and BMP-2 displayed stronger fluorescence signals. The proteins were widely distributed on the BT scaffold surface and overlapped well with the cell spreading areas, while the fluorescence signals in the BTen group were weaker and more dispersed. DAPI staining further showed the distribution of cell nuclei on the scaffold surface. Quantitative analysis of DAPI-positive nuclei indicated a higher number of BMSCs in the BT group than in the BTen group, suggesting that the BT structure provided a more favorable microenvironment for BMSC adhesion and/or proliferation (Fig. S4). To minimize the influence of differences in cell number on the interpretation of osteogenic differentiation, ELISA results were further normalized to the corresponding total cellular protein content measured by the BCA assay (Fig. S5). After normalization, the BT group still showed significantly higher levels of RUNX2, OCN, DCN, and BMP-2 than the BTen group, confirming that the biomimetic trabecular-like structure enhanced osteogenic protein expression at an early stage rather than merely increasing cell abundance [24]. At the transcriptional level, qPCR results revealed temporal changes in the osteogenic phenotype. On day 7, RUNX2, OCN, DCN, and BMP-2 mRNA expression was significantly higher in the BT group compared to that in the BTen group. By day 14, the expression differences remained, with some osteogenic maturation-related genes maintaining higher levels in the BT group, indicating that osteogenic differentiation was more sustained on the BT scaffold (Fig. 6B).

### Postsampling external appearance and x-ray observation

**Fig. 7.**
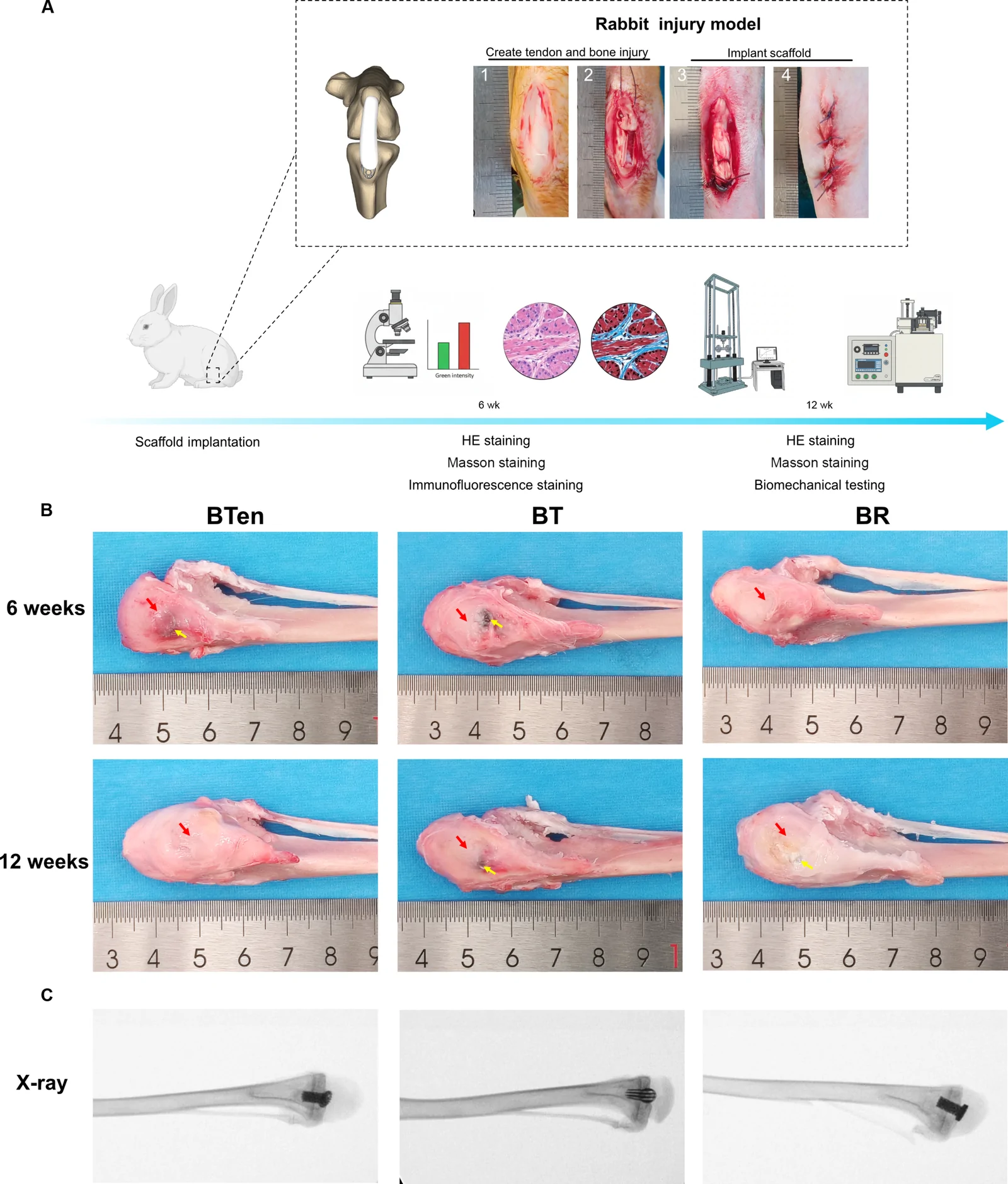
In vivo evaluation of a rabbit patellar tendon acute injury model and implanted scaffolds. (A) Schematic diagram of the in vivo animal experiment procedure. (B) Appearance photos and local magnification images (white dashed box) of the patellar tendon–tibia complex in each group at 6 and 12 weeks postsurgery. Yellow arrows indicate the scaffold region, and red arrows indicate the coverage of newly formed tissue. (C) X-ray images at 12 weeks postsurgery.

To visually assess tissue coverage around different scaffold designs in vivo, specimens from each group were collected at 6 and 12 weeks postsurgery. The external tissue appearance around the prosthesis was recorded, and x-ray imaging was used to observe the overall positioning of the prosthesis (Fig. 7A).

The external appearance results showed that at 6 weeks postsurgery, both the BTen and BR groups exhibited noticeable soft tissue coverage over the top cap region of the prosthesis. The prosthesis was surrounded by continuous soft tissue, with a relatively smooth interface contour. Soft tissue was predominantly concentrated in the top cap region, naturally transitioning with the surrounding tissue (Fig. 7B). In contrast, the BT group showed limited soft tissue coverage in the top cap area, with parts of the prosthesis outline clearly visible and less complete soft tissue encapsulation compared to those of the BTen and BR groups. By 12 weeks postsurgery, these differences remained, with the BTen and BR groups maintaining substantial soft tissue coverage, while the BT group showed limited improvement in soft tissue coverage. X-ray images at 12 weeks revealed that the prostheses in all groups remained in their predetermined position in the proximal tibia, with no evident fractures or structural damage observed.

### Micro-CT evaluation

To assess the impact of different scaffold designs on bone ingrowth in vivo, micro-CT scans were performed on the BTen, BT, and BR groups at 6 and 12 weeks postsurgery. Quantitative analysis of the microstructural parameters of new bone around the prostheses was also conducted.

**Fig. 8.**
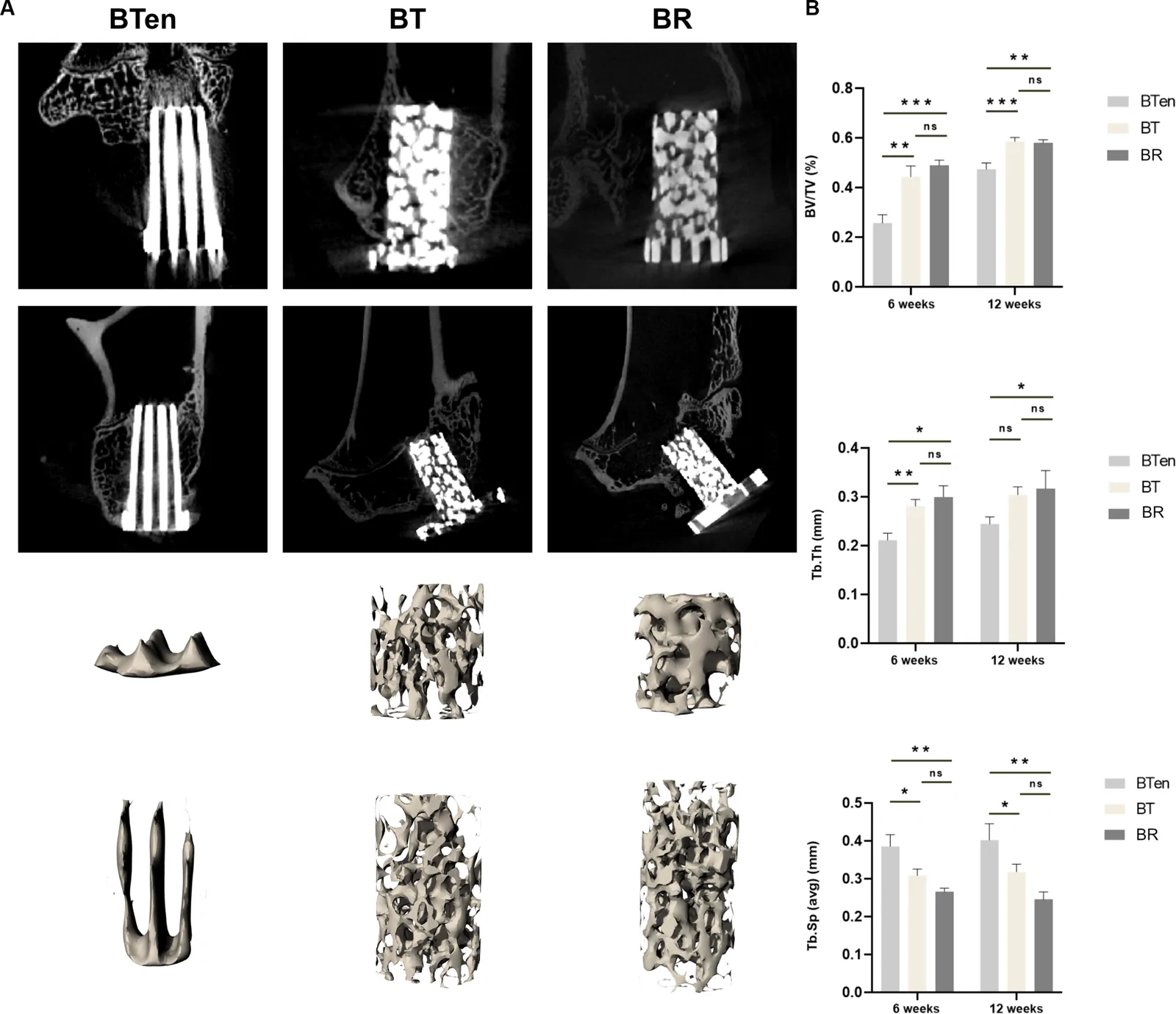
Micro-computed tomography (micro-CT) analysis of new bone at 6 and 12 weeks. (A) Three-dimensional reconstruction images of new bone and scaffolds at 6 and 12 weeks postsurgery. (B) Quantitative evaluation of bone regeneration within the porous scaffolds using bone volume fraction (BV/TV), trabecular thickness (Tb.Th), and trabecular separation (Tb.Sp). ns denotes no statistical significance; *P < 0.05, **P < 0.01, and ***P < 0.001. DG, double-helix structure.

Figure 8A shows that new bone formation was observed around the prostheses in all groups, with distinct differences in the bone ingrowth patterns. In the BTen group, both the top cap and body of the prosthesis were designed with a biomimetic tendon-like orientation structure, with new bone distribution being relatively limited. Bone tissue was primarily concentrated in the peripheral area of the prosthesis, with minimal extension into the internal pores. In contrast, the BT group featured a biomimetic trabecular-like disordered porous structure for both the top cap and body, with new bone extensively distributed on the prosthesis surface and within the internal pores, exhibiting a more continuous bone ingrowth pattern. In the BR group, although the top cap had an ordered structure, the body still adopted a disordered trabecular structure, and the overall bone ingrowth was similar to that of the BT group. Notably, in the 6-week micro-CT scan, some BTen samples showed abnormal prosthesis positions, with varying degrees of extrusion, which was not observed in the BT or BR group. Combined with the imaging results, the BTen group exhibited lower levels of bone ingrowth around the prosthesis, which may be attributed to inadequate early bone–prosthesis interface integration. Quantitative analysis further supported these observations. At 6 weeks postsurgery, the BV/TV in the BT and BR groups was markedly higher than that in the BTen group, with no markedly difference between BT and BR. By 12 weeks, BV/TV increased in all groups, with the BT and BR groups maintaining higher levels, significantly outperforming the BTen group. Tb.Th was also higher in the BT and BR groups, while the BTen group showed relatively lower values. Correspondingly, the Tb.Sp in the BTen group was higher than those in the other 2 groups at both time points (Fig. 8B).

### Histological analysis

To further evaluate the in vivo histological response to different scaffold designs and their temporal evolution, undecalcified hard tissue sections of specimens collected at 6 and 12 weeks postsurgery were stained with HE and Masson’s trichrome for observation.

**Fig. 9.**
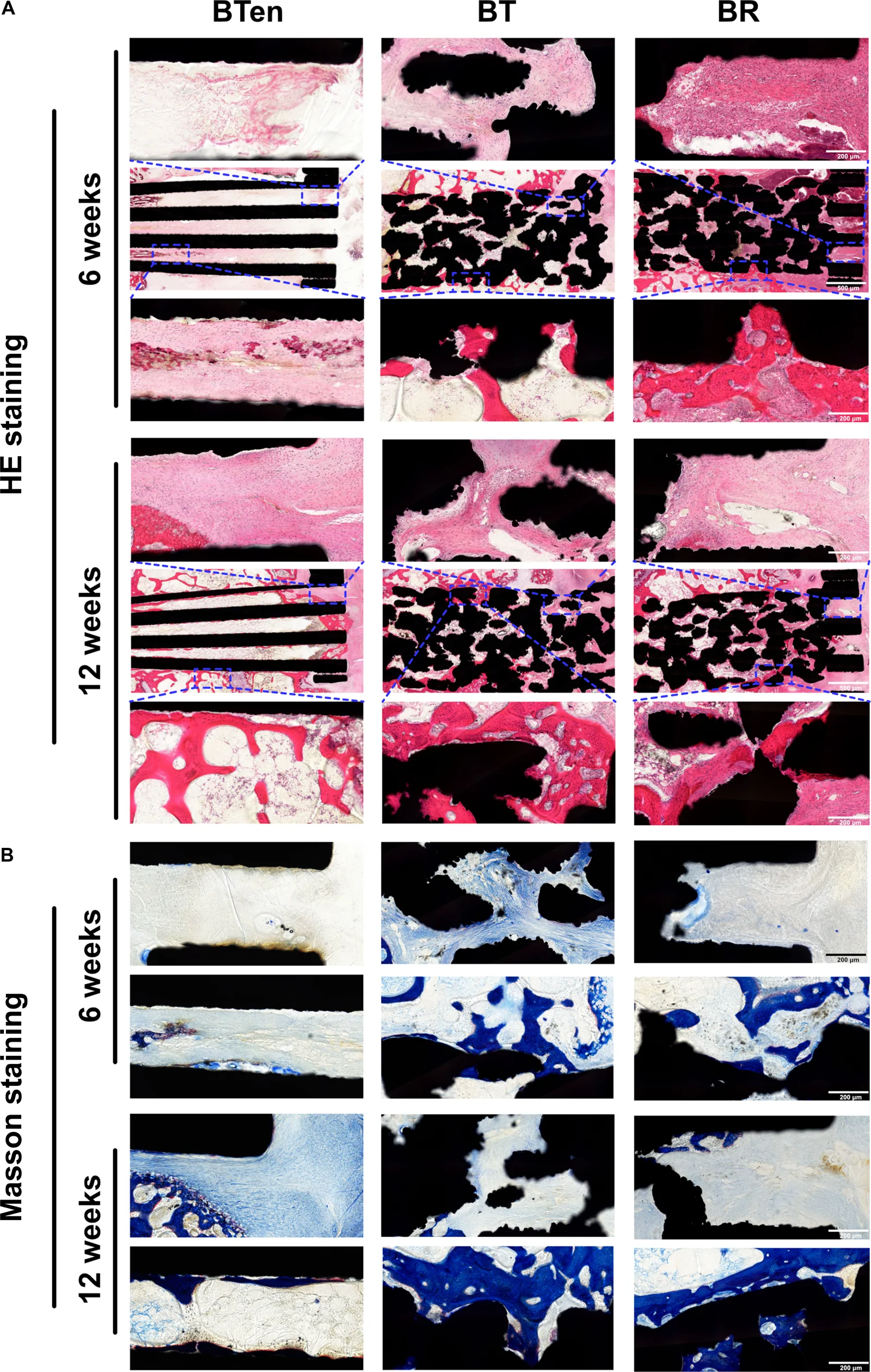
Histological staining evaluation after scaffold implantation in animals. (A) Hematoxylin and eosin (HE) staining of hard tissue sections at 6 and 12 weeks post-scaffold implantation. (B) Masson staining of hard tissue sections at 6 and 12 weeks post-scaffold implantation.

In the HE staining at 6 weeks postsurgery, different degrees of tissue filling were observed around the prostheses, with distinct distribution patterns. In the BTen group, the prosthesis body exhibited a regular orientation, surrounded mainly by fibrous tissue, with limited infiltration into the scaffold’s interior. A relatively clear interface was observed between the prosthesis and surrounding bone tissue. In contrast, in the BT group, more new tissue filled the internal pores of the prosthesis, with irregular tissue distribution extending into the scaffold’s interior. The BR group exhibited regional differences: the top cap area was predominantly covered by continuous fibrous tissue, while in the body region, new tissue infiltrated the disordered pore structure, with a relatively tight bone-tissue interface. By 12 weeks postsurgery, the differences evolved further. In the BTen group, fibrous tissue continued to surround the prosthesis, with a more uniform distribution, although tissue infiltration into the scaffold remained limited. In the BT group, tissue filling in the internal pores increased markedly, with a trabecular-like structure formed, enhancing the continuity of the interface between the prosthesis and the surrounding bone (Fig. 9A). The BR group showed more pronounced tissue ingrowth in the body region, with denser tissue distribution in the pores, while the top cap area maintained a fibrous tissue cover.

Masson staining revealed the distribution of collagen matrix deposition. At 6 weeks postsurgery, in the BTen group, continuous collagen staining was mainly observed around the prosthesis, concentrated on the surface in a sheetlike or bandlike distribution. In the BT and BR groups, collagen staining extended beyond the surface into the pores, forming continuous structures across some pores. By 12 weeks, the BT and BR groups showed markedly increased collagen-rich tissue in the internal pores of the prosthesis, with more continuous collagen distribution forming a trabecular-like network. In the BTen group, collagen deposition remained primarily distributed around the prosthesis (Fig. 9B).

### Immunofluorescence staining

**Fig. 10.**
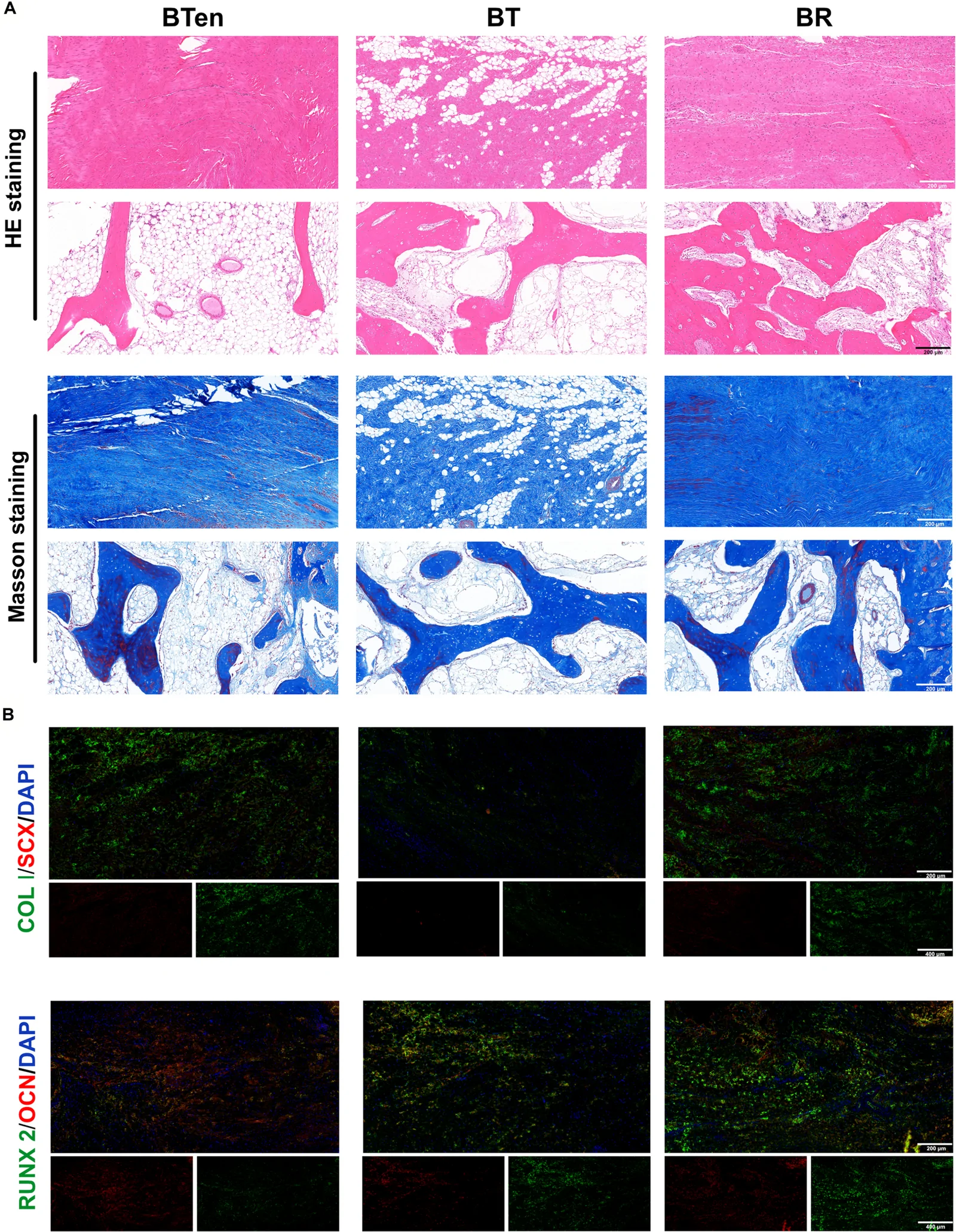
Histological and immunofluorescence evaluation of extracted tissues. (A) Hematoxylin and eosin (HE) and Masson staining of extracted tissues 12 weeks after scaffold implantation. (B) Immunofluorescence staining of tendon markers (collagen I [COL I] and scleraxis [SCX]) and osteogenic markers runt-related transcription factor 2 (RUNX2) and osteocalcin (OCN) in extracted tissues 12 weeks after scaffold implantation.

HE and Masson staining results (Fig. 10A) showed clear regional tissue differences in the top cap and body regions of the prostheses at 6 weeks postsurgery. In the BTen group, the top cap was predominantly covered by continuous dense fibrous tissue, with relatively organized tissue arrangement, while tissue filling in the body region was limited, and bone-like tissue was sparse. In contrast, the BT group showed more trabecular-like bone tissue filling the internal pores of the prosthesis, extending inward, with a tighter bone–prosthesis interface. The top cap in the BT group exhibited a looser fibrous tissue cover. The BR group showed both characteristics, with fibrous tissue covering the top cap and more abundant bone-like tissue filling in the body region. Masson staining further highlighted structure-related differences in collagen matrix deposition. In the BTen group, the top cap showed abundant continuous blue-stained collagen fibers, while collagen deposition in the body region was limited. The BT group exhibited more prominent collagen deposition in the body region, with the collagen matrix distributed along the prosthesis pores, forming a trabecular-like structure, while collagen distribution in the top cap was more dispersed. In the BR group, collagen deposition exhibited clear regional characteristics, with the top cap rich in collagen fibers and the body region showing collagen matrix infiltration into the pores and extending inward.

Immunofluorescence staining at 6 weeks revealed clear regional selectivity in phenotype distribution at the interfaces. In the BTen group, COL I and SCX signals, markers of tenogenic differentiation, were abundant in the top cap and distributed continuously, while osteogenic markers RUNX2 and OCN showed weak expression, mainly limited to the peripheral body region. In contrast, the BT group showed more pronounced RUNX2- and OCN-positive signals in the body region, while COL I and SCX expression was lower in the top cap. The BR group exhibited higher levels of both tenogenic and osteogenic markers in different regions, with COL I and SCX signals in the top cap and RUNX2 and OCN in the body region, reflecting phenotype distribution consistent with the structural regions (Fig. 10B).

### Mechanical pullout testing

To further determine whether the observed tissue integration translated into functional mechanical stability, pullout testing was performed at 6 and 12 weeks after implantation. As shown in Fig. S6, the maximum pullout force increased from 6 to 12 weeks in all groups, indicating progressive maturation of the tendon–prosthesis interface over time. At 6 weeks, the BR group showed a significantly higher maximum pullout force than the BT group, while no significant difference was observed between the BTen and BT groups. At 12 weeks, the BR group exhibited the highest maximum pullout force among all groups, which was significantly higher than those of both the BTen and BT groups. In addition, the BTen group showed a higher maximum pullout force than the BT group at 12 weeks. These results suggest that the region-specific biomimetic design enhanced the functional mechanical stability of the tendon–prosthesis interface in vivo.

## Discussion

Severe bone defects and complex joint reconstruction surgeries often disrupt both the bony support structure and the natural attachment points of soft tissues like tendons, leading to the challenge of achieving integration at both the bone–prosthesis and tendon–prosthesis interfaces, which have distinct biological needs [1]. Although considerable progress has been made with porous titanium alloy prostheses based on 3D-printing technology to promote bone tissue ingrowth and enhance the mechanical stability of the bone–prosthesis interface, the microporous structural design has largely focused on bone integration, neglecting optimization for soft tissue attachment. This has resulted in a mismatch in the integration of heterogeneous interfaces [7]. Due to the high elastic modulus of titanium alloy and its relatively biologically inert surface characteristics, tendons tend to heal on metal prostheses in an unordered scar-like manner, making it difficult to form stable and physiologically functional attachment structures. This is considered a major reason for limited joint function following clinical prosthetic implantation [9]. Previous strategies to improve tendon attachment through surface modification or bioactive factor delivery can regulate interface cellular behavior to some extent, but their release effects are difficult to maintain for long-term stable integration [14]. In this context, this study adopts a region-specific biomimetic design approach, combining biomimetic trabecular bone-like porous structures on the bone side and ordered tendon-like microstructures on the soft tissue side within the same 3D-printed titanium alloy prosthesis. The aim is to provide a unified structural foundation for the synergistic integration of bone–prosthesis and tendon–prosthesis heterogeneous interfaces, offering a new design concept for achieving both mechanical stability and functional soft tissue reconstruction in complex joint reconstructions.

In this study, based on prior research and the practical requirements of different experimental scenarios, 2 scaffold specifications suitable for in vitro and in vivo experiments were designed. Porous Ti6Al4V scaffolds with clear structural features were successfully fabricated using SLM technology. SEM observations revealed that all scaffold groups successfully replicated the intended structural designs. The biomimetic tendon (BTen) scaffold displayed a regular, ordered linear micropore arrangement, while the biomimetic trabecular (BT) scaffold exhibited a highly interconnected, randomly oriented trabecular-like structure, demonstrating the excellent controllability of SLM technology in constructing complex porous metal microstructures [25]. Further measurements of pore size and porosity showed that both scaffolds had an actual pore size of approximately 500 μm and porosity of around 60%, indicating that, while maintaining different biomimetic topological structures, the overall porosity parameters were effectively controlled, minimizing potential interference from pore size or porosity variations in subsequent biological outcomes. From a biological perspective, these pore size and porosity ranges have been widely confirmed to be favorable for cell adhesion, migration, and tissue ingrowth, providing a reasonable foundation for comparing the effects of different microstructures on cellular behavior regulation [19,20]. Additionally, both scaffold surfaces showed some degree of unmelted powder particles, forming micro/nanoscale rough textures that provide advantageous binding sites for protein adsorption and early cell adhesion. EDS analysis revealed that the main elements of the scaffolds were Ti, Al, and V, with mass percentages highly consistent with those of standard Ti6Al4V alloy, and no additional impurity peaks were detected, indicating that the SLM fabrication process was stable and reliable, without introducing detectable chemical contamination.

Despite the similarity in material composition, pore size, and porosity between the BTen and BT scaffolds, marked differences in the growth and alignment of mesenchymal stem cells from different sources were observed on the 2 biomimetic scaffolds, suggesting that microstructural topology plays a crucial role in regulating cell behavior. Specifically, TDSCs, derived from tendon tissue, are naturally exposed to highly anisotropic collagen fiber bundles in the physiological environment, which leads to their elongated spindle shape and axial alignment along the fiber direction [26,27]. The ordered linear micropores and streamlined framework of the BTen scaffold provide typical contact guidance signals, making it easier for TDSCs to polarize morphologically, form pseudopodia oriented along the main axis, and spread directionally. The clear F-actin stress fibers and consistent orientation observed in the staining results further support this phenomenon. Studies have shown that cytoskeletal polarization is often accompanied by more stable focal adhesion assembly (integrin clustering and focal adhesion kinase [FAK] activation) and enhanced cytoskeletal tension, with sufficient early adhesion and spreading being a prerequisite for increased cell proliferation [28,29]. This coupling relationship between structure, morphology, and cell proliferation helps explain the persistent proliferation advantage of the BTen scaffold in the TDSC co-culture system. In contrast, the disordered trabecular-like network of the BT scaffold, while exhibiting higher spatial complexity, lacks a clear dominant orientation, causing TDSCs to exhibit dispersed morphology and insufficient polarization on the multidirectional curved surfaces and interlaced supporting structures, which limits their spreading efficiency and the rapid establishment of intercellular connections, ultimately leading to lower overall proliferative activity. Meanwhile, BMSCs show a structural preference that more closely resembles natural bone characteristics [30]. The disordered interconnected trabecular network of the BT scaffold, with its microstructural complexity, curvature surfaces, beam–column junctions, and abundant 3D adhesion sites, facilitates BMSCs’ 3D migration, interpore bridging, and collective network formation within the pores [31]. The dense, interwoven F-actin network formed by BMSCs on the BT scaffold reflects an active cytoskeletal remodeling process in 3D space. For mesenchymal stem cells, such 3D interconnected structures can induce mechanical sensory states for osteogenesis, with the heterogeneous mechanical environment at the nodes and beam–column areas enhancing focal adhesion maturity and cytoskeletal tension, activating the mechanotransduction pathways related to cell proliferation and osteogenic potential [32]. In contrast, the linear ordered structure of the BTen scaffold favors a restricted adhesion path with ordered alignment, limiting the 3D spatial occupation and intercellular network formation of BMSCs, leading to lower overall expansion efficiency compared to the BT group. From the perspective of scaffold biocompatibility, permeability represents an important architecture-dependent parameter because it affects nutrient exchange, waste removal, and the local fluid microenvironment for cell survival and growth. The higher relative permeability of the BT structure may partly explain its stronger support for BMSC proliferation and 3D network formation, whereas the biological advantage of the BTen structure for TDSCs appears to rely more on contact guidance and ordered cytoskeletal alignment [33]. This result, at the cellular behavior level, validates the rationale for applying biomimetic tendon and trabecular structures at the soft tissue and bone interfaces and provides a foundation for integrating different biomimetic microstructures within the same metal prosthesis to achieve directed differentiation and regulation of cell behavior at the heterogeneous bone–prosthesis and tendon–prosthesis interfaces.

Based on the differences in biocompatibility and scaffolding, this study further revealed the significant impact of different biomimetic microstructures on cell fate determination from the perspective of phenotypic differentiation. In the tenogenic differentiation assessment, the BTen scaffold significantly enhanced the expression of tenogenic-related phenotypic markers in TDSCs at multiple time points. Immunofluorescence, ELISA, and RT-qPCR results consistently showed that key tenogenic markers such as COL I, SCX, TNMD, and TNC were continuously up-regulated in the BTen group. The relevant proteins were distributed along the cell alignment direction, forming a typical tendon-like tissue structure. COL I, as the main structural protein of the tendon extracellular matrix, was stably and highly expressed, indicating that the BTen scaffold promotes tendon-like matrix deposition and organization [34]. SCX, a key transcription factor for tendon lineage commitment, was significantly up-regulated early in culture, suggesting that the biomimetic tendon structure effectively triggers TDSCs to undergo tenogenic differentiation. Furthermore, the sustained increase in TNMD and TNC in the later stages reflects the progression of the tendon-related phenotype from activation to maturation [35]. Combined with the aforementioned cell morphology and F-actin staining results, it can be inferred that the ordered anisotropic microstructure of the BTen scaffold promotes pronounced morphological polarization and directional alignment of TDSCs through contact guidance, thereby enhancing cytoskeletal tension and stabilizing focal adhesion structures [36]. Previous studies have shown that mechanical stimuli induced by such structures can activate FAK signaling through integrin-mediated focal adhesions, further regulating the nuclear translocation of Yes-associated protein (YAP) and transcriptional coactivator with PDZ-binding motif (TAZ), ultimately influencing the initiation and maintenance of tendon lineage-related transcriptional programs [37]. The sustained high expression of SCX suggests that the integrin–FAK–YAP/TAZ–SCX axis may be involved in the tenogenic differentiation process mediated by the biomimetic tendon structure.

In contrast, the BT scaffold exhibited marked advantages in promoting BMSC osteogenic differentiation. From the early increase in ALP activity and mineralized nodule formation to the sustained up-regulation of osteogenic proteins and gene expression, the BT group consistently outperformed the BTen group at different time points, indicating that the biomimetic trabecular structure effectively promotes and maintains osteogenic differentiation. The increase in ALP activity reflects enhanced matrix maturation and mineralization readiness in the early stages of osteogenic differentiation [38], while the marked increase in the number and density of mineralized nodules in ARS staining shows that mineral deposition was more fully advanced on the BT scaffold. At the molecular level, the significant up-regulation of RUNX2, a core transcriptional regulator of the osteogenic lineage, in the BT group suggests that this structure favors early commitment to osteogenic differentiation. The sustained high expression of OCN and DCN further indicates the stable progression of bone-like matrix synthesis and mineralization [39]. The enhanced expression of BMP-2 in the BT group suggests that the biomimetic trabecular structure not only regulates cell behavior through the mechanical microenvironment but may also play a synergistic amplifying role in osteogenic signaling networks [40]. Structurally, the 3D interconnected trabecular-like network of the BT scaffold more closely resembles natural cancellous bone at the microscale. Its curved surfaces and beam–column junctions provide abundant 3D adhesion sites and a heterogeneous mechanical environment [41], which has been shown to promote focal adhesion maturation and enhance RhoA/ROCK-mediated cytoskeletal tension, thus activating the FAK and its downstream integrin–FAK–RUNX2 osteogenic regulatory axis, consistent with the trends in osteogenic marker expression observed in this study [42].

Micro-CT and histological analyses further revealed (Figs. 8 and 9) that different biomimetic structural designs markedly affected the stability of the bone–prosthesis interface and the bone ingrowth pattern in vivo, with these differences becoming apparent in the early postoperative period and gradually amplifying over time. Micro-CT 3D reconstruction and quantitative analysis showed that both the BT and BR groups exhibited higher BV/TV and Tb.Th and lower Tb.Sp at both 6 and 12 weeks postsurgery. New bone was not only distributed on the prosthesis surface but also extended into the internal porous structure, whereas the BTen group showed new bone primarily limited to the peripheral region of the prosthesis, with limited extension into the internal pores, resulting in significantly lower bone volume fraction and Tb.Th. Notably, the BTen group exhibited a trend of prosthesis extrusion in some specimens at 6 weeks postsurgery, suggesting that insufficient early bone–prosthesis interface integration may lead to increased micromotion at the interface, thus affecting overall stability [43]. Previous studies have shown a close coupling between insufficient bone ingrowth and interface micromotion, where early mechanical instability can inhibit osteoblast adhesion and mineralization, forming a negative feedback loop detrimental to prosthesis fixation, which aligns well with the quantitative results and this consensus [44,45]. From a structural perspective, the biomimetic trabecular-like disordered porous structure (in the osteogenic regions of BT and BR) more closely resembles the 3D topological shape of natural cancellous bone. Its high connectivity, curved surfaces, and beam–column–node junctions provide abundant 3D adhesion sites and a heterogeneous mechanical environment, which promotes vascular invasion, cell migration, and mineralized matrix deposition [46]. This structural advantage was further confirmed at the histological level. HE and Masson staining showed that in the BT and BR groups, a large amount of trabecular-like bone tissue filled the prosthesis pores in the body region at 12 weeks postsurgery, with continuous collagen matrix distribution and a tighter bone–prosthesis interface. In the BTen group, tissue filling was still predominantly fibrous tissue around the prosthesis, with limited infiltration into the internal structure and relatively insufficient interface continuity. The BR group showed the highest maximum pullout force, particularly at 12 weeks, indicating that the region-specific biomimetic design translated tissue-level integration into improved mechanical stability at the tendon–prosthesis interface. This improvement may be attributed to the coordinated structural partitioning of the BR prosthesis, with the tendon-like ordered top cap promoting soft tissue attachment and the trabecular-like porous body supporting bone ingrowth and stable fixation.

Immunofluorescence results further supported the histological differences at the molecular phenotype level, as shown in Fig. 10, suggesting that different biomimetic structures shape localized mechanical and cellular microenvironments, thereby driving differentiated regenerative processes. At 6 weeks postsurgery, RUNX2- and OCN-positive signals in the osteogenic regions of the BT group were markedly enhanced, indicating that osteogenic transcriptional regulation and mineralization maturation processes dominate in this area, consistent with more extensive bone ingrowth into the pores [24,47]. In contrast, in the BTen group, osteogenic marker signals were primarily confined to the periphery of the prosthesis, with relatively low expression levels within the osteogenic region, suggesting that bone migration into the deeper pores and mineralized matrix deposition may be limited. This difference not only reflects the different cell differentiation lineages but may also be related to the density of integrin adhesion sites and the fluid shear environment determined by the 3D topological structure. The biomimetic trabecular-like disordered porous structure typically has higher connectivity and curved surfaces, providing more abundant 3D adhesion interfaces and creating a heterogeneous mechanical stimulus closer to that of natural cancellous bone, which promotes the activation of adhesion-related signaling and the maintenance and progression of the osteogenic phenotype [48]. In contrast, the more orientation-guided structural characteristics are advantageous at the soft tissue interface, but without the 3D mechanical stimulation spectrum matching that of cancellous bone at the bone interface, they may hinder the establishment of deep trabecular ingrowth [49]. The key value of the BR group lies in the synergy of the region-specific biomimetic structure, which forms relatively independent and complementary regenerative microenvironments within the same prosthesis, reducing the mechanical conflict between bone fixation at the load-bearing interface and functional soft tissue attachment. The top cap region is predominantly covered by fibrous tissue, with richer COL I and SCX signals, meeting the soft tissue’s need for oriented adhesion and collagen assembly; the prosthesis osteogenic region retains the biomimetic trabecular disordered porous topology, with predominant RUNX2- and OCN-positive expression, and bone-like tissue extensively enters the pores, achieving an overall bone ingrowth level close to that of the BT group. The histological and molecular phenotype distribution of these spatial partitions corresponds to the prosthesis microstructure partition, suggesting that region-specific biomimetic design can regulate cell adhesion and mechanical stimulation spectra through regional control, establishing local microenvironments favorable for tenogenesis and osteogenesis in vivo, thereby promoting the synergistic integration of heterogeneous interfaces.

In summary, a single-interface biomimetic strategy is insufficient to simultaneously meet the dual-interface requirements of soft tissue attachment and bone fixation stability. Biomimetic tendon-like ordered structures promote soft tissue phenotype formation but have limited bone ingrowth capacity, while biomimetic trabecular structures significantly enhance bone integration but fail to optimize the soft tissue attachment environment. By incorporating a region-specific biomimetic design with structural partitioning within the same prosthesis, the processes of tenogenesis and osteogenesis can be independently regulated in different regions, leading to more coordinated and stable heterogeneous dual-interface tissue integration in vivo. This finding systematically validates the heterogeneous dual-interface synergistic integration design concept proposed in this study, providing a design approach with clear biological evidence and translational potential for prosthesis structure optimization in complex bone defects and joint reconstruction.

## Conclusion

This study proposes and validates a 3D-printed region-specific biomimetic titanium alloy prosthesis design strategy, addressing the biological integration requirements of heterogeneous bone–prosthesis and tendon–prosthesis double interfaces in complex bone defect joint reconstructions. By integrating biomimetic tendon-like ordered microstructures and biomimetic trabecular-like disordered porous structures into distinct functional regions within the same Ti6Al4V prosthesis, this study systematically elucidates the regulatory effects of biomimetic microporous structures on cell behavior, lineage differentiation, and interface tissue integration patterns. In vitro experiments showed that the biomimetic tendon-like structure markedly promoted the directional alignment and tenogenic differentiation of TDSCs, while the biomimetic trabecular-like structure was more beneficial for BMSC osteogenic differentiation and mineralization maturation, revealing lineage-specific differentiation characteristics mediated by the structure. In vivo animal experiments further confirmed that a single structure is insufficient to simultaneously meet the dual requirements of soft tissue attachment and bone fixation stability, while the region-specific biomimetic design can create region-specific tenogenic and osteogenic microenvironments in vivo, achieving more coordinated heterogeneous double-interface tissue integration. This study systematically validates the advantages of region-specific biomimetic structures in multi-tissue interface reconstruction at the cellular, tissue, and in vivo levels, providing a theoretical basis for the structural optimization of prostheses in complex bone defect and joint reconstructions.

## Data Availability

The data that support the findings of this study are available from the corresponding authors upon reasonable request.

## Supplementary Materials

Supplementary 1 Graphical Abstract Supplementary Text Figs. S1 to S6 Tables S1 and S2
