# Supplementary material for: 3D-Printed Region-Specific Biomimetic Prosthesis for Enhancing Heterogeneous Double-Interface Integration of Bone–Prosthesis and Tendon–Prosthesis
Source: Biomater Res. 2026 Sep 11;30:0416. doi: 10.34133/bmr.0416 (PMC13562796; doi:10.34133/bmr.0416)
Supplement: Supplementary file 1 [file bmr.0416.f1.docx]

**Supplementary material:**

**3D Printed Region-Specific Biomimetic Prosthesis for Enhancing Heterogeneous Double-Interface Integration of Bone-Prosthesis and Tendon-Prosthesis**

**1. Extraction, Culture, and Identification of TDSCs and BMSCs**

Tendon-derived stem cells (TDSCs) were isolated from the patellar and Achilles tendons of 3-week-old male rats. After digestion with type I collagenase (BioFrox, Germany) overnight and centrifugation, the cells were resuspended in a basal culture medium containing 10% fetal bovine serum (v/v) and 1% penicillin/streptomycin, then cultured at 37°C in a 5% CO_2_ atmosphere until the third generation. Bone marrow-derived mesenchymal stem cells (BMSCs) were isolated from male rat bone marrow and cultured in DMEM/F-12 (Cytiva, USA) supplemented with 10% fetal bovine serum (HyClone, USA) and 1% streptomycin-penicillin (Gibco, USA) [22]. For identification, the third-generation TDSCs and BMSCs were dissociated into single-cell suspensions, washed with PBS, and adjusted to 1×10^6^ cells/100 μl. After incubating with antibodies targeting CD31, CD44, CD45, and CD90 for 30 minutes at 37°C, flow cytometry was performed to analyze cell surface markers.The multi-lineage differentiation potential of TDSCs and BMSCs was assessed by inducing osteogenesis, adipogenesis, and chondrogenesis. For osteogenesis, Alizarin Red S staining was used to detect calcium deposition after 3 weeks of induction. For adipogenesis, lipid droplet formation was observed using Oil Red O staining after 3 weeks of induction. For chondrogenesis, a chondrogenic staining kit was used to assess differentiation after 3 weeks of induction. All differentiation experiments were performed using the same protocols for both TDSCs and BMSCs.

**2. Identification of Isolated Cells**

After the third passage, the isolated BMSCs and TDSCs exhibited typical spindle-shaped adherent growth with organized cell arrangement, uniform morphology, and good confluence, which are characteristic of mesenchymal stem cells (MSCs). To further identify the cell phenotype, flow cytometric analysis of surface markers was performed for both types of MSCs. **Fig. S1** shows that both BMSCs and TDSCs highly expressed MSC-related markers CD44 and CD90, with positive expression rates exceeding 90%. CD44, an important glycoprotein mediating cell-extracellular matrix interactions, and CD90, a widely used MSC marker, were both highly expressed. In contrast, the endothelial cell marker CD31 and hematopoietic cell marker CD45 showed expression rates of less than 5%, consistent with previous reports, indicating high purity of the isolated cell populations with minimal contamination from other cell types. To assess the multi-lineage differentiation potential, both BMSCs and TDSCs were induced for osteogenesis, chondrogenesis, and adipogenesis. After 3 weeks of induction, Alizarin Red S staining revealed clear red calcium nodules, indicating good osteogenic differentiation [22]. Oil Red O staining showed scattered lipid droplets within the cells, suggesting adipogenic potential. Under chondrogenic induction conditions, Alcian Blue staining showed clustered cells with significant blue matrix deposition, confirming chondrogenic differentiation potential. These results demonstrate that both cell types possess the typical tri-lineage differentiation potential of MSCs. Additionally, to evaluate the functional behavior of TDSCs and BMSCs, a scratch wound healing assay was performed. The results showed that both TDSCs and BMSCs exhibited migratory ability, with significant cell coverage of the wound area after 24 hours, indicating good migration and wound-healing potential. Based on morphology, surface marker expression, multi-lineage differentiation potential, and functional behavior, the cells isolated in this study displayed typical biological characteristics of BMSCs and TDSCs, with high purity and good functional activity.

**Table.S1** Primers used in Real-Time PCR for osteogenesis-related genes

| Gene | Forward primer sequence (5′-3′) | Reverse primer sequence (5′-3′) |
| --- | --- | --- |
| β-Actin  COL Ⅰ | GACAGGATGCAGAAAGAG  CTGGCAACCTCAAGAAGTCC | ACATGATCTGGGTCATGAG  CAAGTTCCGGTGTGACTCG |
| SCX  TNMD | CTTCGCCATGCTGCGTTC  TGGAGATTTGCGACAATGTGAC | AGCATGAACACGACAGGGTT  GGGACCACCCATTGCTCATT |
| TNC  RUNX2 | TCTGCAGGAGGACATCAAA  AAGGAGGAGCAGAGGGAAGG | AAGGAGTTCAGGAGAGGAGG  AGGAGGATGGAGGGGAAGAA |
| OCN | ACAGCAGACACAGGAGGAC | CTCAGGAGCAGAGGAGGAG |
| DCN | AGAGGAGGAAAGCAGAGAGG | TTTCCTTCCAGGAGGAGGAG |
| BMP-2 | TCCAGGAGGAGAGGAGAAAG | GGAAGGGGAAGGAGAGGAGG |

COL Ⅰ:type I collagen, TNMD:Tenomodulin;SCX:Scleraxis; TNC:Tenascin C; RUNX2: Runt-related transcription factor 2; OCN : Osteocalcin DCN : DecorinBMP-2 : Bone Morphogenetic Protein 2

**Table S2. Morphological parameters of SLM-fabricated porous scaffolds based on CAD design and micro-CT measurements.**

| Scaffold | Porosity(%) | |  | Pore size (μm) | |
| --- | --- | --- | --- | --- | --- |
| Name | Designed porosity | Micro-CT-measured |  | Designed pore size | Micro-CT-measured |
| BTen | 60 | 56.8 ± 1.9 |  | 500 | 472 ± 29 |
| BT | 60 | 54.6 ± 2.4 |  | 500 | 459 ± 35 |

**Figure. S1.Quantitative pore size distribution of the biomimetic trabecular scaffold.**

**Figure. S2.** **Characterization of tendon-derived stem cells (TDSCs) and bone marrow–derived mesenchymal stem cells (BMSCs).** (A) Flow cytometric analysis of TDSCs showing the expression of CD31, CD44, CD45, and CD90. (B) Scratch wound assay images of TDSCs at 0 and 24 h, demonstrating cell migration. (C) Osteogenic, chondrogenic, and adipogenic differentiation of TDSCs. (D) Flow cytometric analysis of BMSCs showing the expression of CD31, CD44, CD45, and CD90. (E) Scratch wound assay images of BMSCs at 0 and 24 h. (F) Osteogenic, chondrogenic, and adipogenic differentiation of BMSCs.

**Fig.S3. CFD-based permeability analysis of BTen and BT structures.** (A) Relative permeability of BTen and BT structures normalized to the BT group. (B) Pressure difference under identical flow conditions. (C) Wall shear stress distribution. (D) Representative CFD visualizations showing pressure distribution, velocity field, velocity vector distribution, and wall shear stress distribution in BT and BTen structures.

**Fig.S4. ImageJ-based quantification of DAPI-positive nuclei.** DAPI-positive nuclei of TDSCs and BMSCs cultured on BTen and BT scaffolds were quantified from 20 randomly selected fields per group. Data are presented as violin plots with median and quartiles. *p < 0.05, ***p < 0.001.

**Fig.S5. Quantification of total cellular protein concentration by BCA assay.** Total cellular protein concentrations of TDSCs and BMSCs cultured on BTen and BT scaffolds were measured using a BCA protein assay after 7 days of co-culture. For TDSCs, the BTen group showed a higher total cellular protein concentration than the BT group. For BMSCs, the BT group showed a higher total cellular protein concentration than the BTen group. These results were used for normalization of ELISA data to minimize the influence of differences in cell number among scaffold groups. Data are presented as mean ± SD. *p < 0.05, **p < 0.01.

**Fig.S6. Functional mechanical evaluation of the tendon-prosthesis interface.**(A) Representative image showing the pull-out test setup for evaluating the mechanical stability of the tendon-prosthesis interface. (B) Quantitative analysis of the maximum pull-out force in the BTen, BT, and BR groups at 6 and 12 weeks after implantation. Data are presented as mean ± SD. ns, not significant; *p < 0.05, **p < 0.01, ***p < 0.001.
